# Prediction of Subclinical and Clinical Multiple Organ Failure Dysfunction in Breast Cancer Patients—A Review Using AI Tools

**DOI:** 10.3390/cancers16020381

**Published:** 2024-01-16

**Authors:** Andreea-Iuliana Ionescu (Miron), Dimitrie-Ionut Atasiei, Radu-Tudor Ionescu, Flavia Ultimescu, Andrei-Alexandru Barnonschi, Alexandra-Valentina Anghel, Cătălin-Alexandru Anghel, Ionuț-Lucian Antone-Iordache, Ruxandra Mitre, Alexandra Maria Bobolocu, Andreea Zamfir, Horia-Dan Lișcu, Simona Coniac, Florica Șandru

**Affiliations:** 1 Department of Oncological Radiotherapy and Medical Imaging, “Carol Davila” University of Medicine and Pharmacy, 020021 Bucharest, Romania; andreea-iuliana.miron@drd.umfcd.ro (A.-I.I.); andrei.barnonschi@stud.umfcd.ro (A.-A.B.); alexandra-valentina.anghel@stud.umfcd.ro (A.-V.A.); catalin.anghel@stud.umfcd.ro (C.-A.A.); antoneiordachelucian@stud.umfcd.ro (I.-L.A.-I.); ruxandra.mitre@drd.umfcd.ro (R.M.); alexandra.c.bobolocu@stud.umfcd.ro (A.M.B.); andreea.zamfir@stud.umfcd.ro (A.Z.); horia-dan.liscu@drd.umfcd.ro (H.-D.L.); 2 Department of Medical Oncology, Colțea Clinical Hospital, 030167 Bucharest, Romania; simona.horlescu@drd.umfcd.ro; 3 Department of Computer Science, University of Bucharest, 010041 Bucharest, Romania; radu.ionescu@fmi.unibuc.ro; 4 Department of Pathology, Institute of Oncology “Prof. Dr. Alexandru Trestioreanu”, 022328 Bucharest, Romania; flavia.ultimescu@drd.umfcd.ro; 5 Department of Pathological Anatomy, “Carol Davila” University of Medicine and Pharmacy, 020021 Bucharest, Romania; 6 Department of Radiotherapy, Colțea Clinical Hospital, 030167 Bucharest, Romania; 7 Department of Endocrinology, “Carol Davila” University of Medicine and Pharmacy, 020021 Bucharest, Romania; 8 Department of Dermatovenerology, “Carol Davila” University of Medicine and Pharmacy, 020021 Bucharest, Romania; florica.sandru@umfcd.ro; 9 Department of Dermatology, Elias University Emergency Hospital, 011461 Bucharest, Romania

**Keywords:** breast cancer, multiple organ failure syndrome, subclinical multiple organ failure, artificial intelligence, quality of life

## Abstract

**Simple Summary:**

The paper discusses precursor lesions of breast cancer and the subclinical syndrome of multiple organ failure, representing early disease stages with changes occurring before symptoms become clinically detectable. It aims to address why breast cancer patients deteriorate and how their condition progresses from subclinical organ failure to acute multiple organ failure. The review utilized databases like PubMed, Google Scholar, and Scopus, focusing on keywords related to organ failure, breast cancer, and quality of life. It emphasizes the need to identify and prevent the onset of multiple organ failure in cancer patients early on to enhance their quality of life and survival. Despite a subclinical organ failure syndrome undetectable by current diagnostics, the text proposes the increasing importance of AI in aiding physicians in diagnosing this syndrome in the future.

**Abstract:**

This review explores the interconnection between precursor lesions of breast cancer (typical ductal hyperplasia, atypical ductal/lobular hyperplasia) and the subclinical of multiple organ failure syndrome, both representing early stages marked by alterations preceding clinical symptoms, undetectable through conventional diagnostic methods. Addressing the question “Why patients with breast cancer exhibit a tendency to deteriorate”, this study investigates the biological progression from a subclinical multiple organ failure syndrome, characterized by insidious but indisputable lesions, to an acute (clinical) state resembling a cascade akin to a waterfall or domino effect, often culminating in the patient’s demise. A comprehensive literature search was conducted using PubMed, Google Scholar, and Scopus databases in October 2023, employing keywords such as “MODS”, “SIRS”, “sepsis”, “pathophysiology of MODS”, “MODS in cancer patients”, “multiple organ failure”, “risk factors”, “cancer”, “ICU”, “quality of life”, and “breast cancer”. Supplementary references were extracted from the retrieved articles. This study emphasizes the importance of early identification and prevention of the multiple organ failure cascade at the inception of the malignant state, aiming to enhance the quality of life and extend survival. This pursuit contributes to a deeper understanding of risk factors and viable therapeutic options. Despite the existence of the subclinical multiple organ failure syndrome, current diagnostic methodologies remain inadequate, prompting consideration of AI as an increasingly crucial tool for early identification in the diagnostic process.

## 1. Introduction

Multiple organ failure dysfunction (MODS), previously known as multiple systemic organ failure (MSOF), represents a life-threatening condition. It is characterized by a dysfunction of two or more organ systems caused at a molecular level by cellular, humoral, and biochemical mediators, which will finally lead to the formation of tissue-damaging end products [1]. Evidence suggests that MODS is among the most important factors that determine the survival rate in patients with advanced cancer in ICU settings, and therefore, caution should be taken.

In patients with an underlying malignancy or early stage of disease, a subclinical syndrome of multiple failure organs can occur—a loss of function of vital organs, potentially reversible, compensated by the organism with its homeostasis. In the context of breast cancer, the exploration of subclinical multiple organ failure syndrome is of clinical significance because it can lead to understanding the underlying mechanisms of disease progression and clinical degradation. Ideally, the subclinical phase of breast cancer requires a comprehensive examination before the onset of clinical manifestations. Therefore, investigating this syndrome in breast cancer patients is imperative for early detection, intervention, and personalized patient management. Unveiling this subclinical phenomenon also provides a unique opportunity to refine treatment strategies because mastectomy, especially when performed on both breasts, is a well-known QoL-reducing factor described in the literature. Thus, our study contributes to improved patient outcomes, prolonged survival, and enhanced quality of life. Despite substantial research, data about the subclinical multiple organ failure syndrome are scarce, especially in oncologic patients. Moreover, this concept is not fully understood by oncologists because of its similar manifestations with adverse reactions of intensive antineoplastic therapies (surgery, chemotherapy, radiotherapy, corticosteroids), oncologic emergencies of sepsis, and disseminated intravascular coagulation [1]. Due to this lack of research, neither treatment nor intervention has proven efficient in preventing or controlling the inadequate response of the body in multiple organ dysfunction syndrome [2].

Recent large clinical studies have turned their attention to adjuvant therapies to limit the metastasis process, but their effect on how they restrain the tumor from disseminating is still unclear [3]. Despite these results, the actual treatment aims at molecular targets like HER2/neu [4] and CDK4/6 inhibitors [5]. However, these key components in molecular aberrations in cancerous cells have yet to be proven as a significant factor in the process of dissemination [6]. Thus, high-quality clinical studies made the actual guidelines for breast cancer therapy and the probability of metastasis conditioned by molecular markers and predictive statistical models [7].

As much as prolonging survival, quality of life (QoL) in oncologic patients should not only be carefully monitored from the beginning of the diagnosis but also improved during the disease and treatment [8,9,10]. Moreover, the European Society of Medical Oncology (ESMO) included health-related quality of life as a parameter of anticancer therapy value by establishing the Magnitude of Clinical Benefit Scale (MCBS) [11]. During the latest period, the quality of life in breast cancer patients was intensively studied; simple but effective strategies like physical activity and psychosocial intervention can considerably improve patients’ QoL. However, complaints such as pain, worry, lymphedema, and sexual dysfunction secondary to treatment have to be monitored more carefully [12]. Cognitive and emotional aspects of life are impaired significantly for patients and their relatives. A breast cancer diagnosis leads to anxiety and depression due to disease-related psychological stress, a poor prognosis, and the imminent loss of a dear member of the family or friend. Thus, the correct management of the symptoms and the comfort of the patient become mandatory.

There is an urgent need to establish a comprehensive, multidisciplinary approach to enhancing the quality of life for breast cancer patients. Palliative and psychological support programs are essential to aiding the patient during and after the treatment ends. Moreover, using QoL scales both in research and clinical practice, we can understand more deeply the impact of disease and treatment on the physical, cognitive, social, and psychological aspects of a patient’s life. EORTC QOQ-C30 is a standardized and validated questionnaire designed by the European Organization for Research and Treatment of Cancer to evaluate the quality of life in oncologic patients. It is composed of several questions that cover different domains, such as general health status, cancer-specific symptoms, cognitive and physical functioning, and general quality of life. EORTC QLQ-BR45 represents an extension of EORTC QOQ-C30, specially designed for the evaluation of breast cancer patients; it covers questions related to breast cancer, such as pain and discomfort in the breast region, body image perception, sexual function, and hair loss. Hence, investigating the quality of life of breast cancer patients with multiple organ failure significantly influences the provision of care and treatment for these patients. This influence manifests through the implementation of personalized and patient-oriented management strategies.

This review aims to investigate the following research question: “Why do patients with breast cancer exhibit a tendency to deteriorate?” It further explores the progression from the subclinical multiple organ failure syndrome into an acute (clinical) state of multiple organ failure, resembling a cascading waterfall or domino effect, wherein the likelihood of the patient reaching the end of life is notably high. Moreover, this research will focus on the epidemiology, pathogenesis, clinical manifestations, and management of MODS in breast cancer patients. The identification and prevention of the onset of the multiple organ failure cascade from the inception of malignancy represent a crucial objective aimed at enhancing the quality of life and prolonging survival in these patients. This effort enhances our understanding of risk factors and explores potential therapeutic options.

## 2. Materials and Methods

The current review is designed as a narrative review. The main objective is to provide a new perspective on multiple organ failure and subclinical organ failure syndrome in oncologic patients, especially breast cancer patients. Additional objectives, such as summarizing the current state of knowledge and identifying the actual gaps in the literature about epidemiology, pathogenesis, and management of multiple organ failure in cancer patients, as well as the quality of life during malignancy, are studied. Research of the literature was performed utilizing databases such as PubMed, Google Scholar, and Scopus during October 2023; “MODS”, “SIRS”, “sepsis”, “pathophysiology of MODS”, “MODS in cancer patients”, “multiple organ failure”, “risk factors”, “cancer”, “ICU”, “quality of life”, and “breast cancer” were used as keywords in the abovementioned search engines, and supplementary references were extracted from the returned articles. No formal inclusion and exclusion criteria were used due to the nature of not being a systematic review. Nonetheless, we used references that covered the concept of MODS, from epidemiology to management, in critically ill patients as well as in cancer patients. Moreover, studies approaching the quality of life of oncologic patients and breast cancer patients in non-intensive care units, as well as in critical care settings, were included.

Artificial intelligence (AI) techniques are often used to group (cluster) items [13], such that items within each group are more similar to each other than to items from other groups [14,15,16]. To discover such groups of research articles on multiple organ dysfunction syndrome (MODS), we conducted an AI-based analysis of the surveyed articles. More specifically, we apply a hierarchical clustering approach [17] to the collected documents. Following Soviany et al. [18], we take into account the titles and abstracts of the articles and apply an agglomerative clustering method based on Ward linkage [19]. This method starts with the individual articles and then gradually merges them into larger groups based on the similarities shared between the articles. The algorithm is executed until all articles are merged into one large group. Ward linkage is a method to decide which groups to merge at each step. The idea is to bring together groups that minimize the increase in total variance or, in simpler terms, minimize the overall entropy when they are merged. In order to estimate the total variance, the method needs a way to compute the similarity between two groups of articles. Soviany et al. [18] used the classical term frequency–inverse document frequency (TF-IDF) scheme to obtain a vectorial representation for each article in their survey. However, Bidirectional Encoder Representations from Transformers (BERT) [20] represent a more recent approach that was found to produce significantly better results across a wide variety of natural language processing tasks. We thus consider that BERT is a more suitable choice for our text clustering task. To compute the similarity, we propose to employ the cosine similarity on top of the class token *[CLS]* extracted by a pre-trained BERT model. We specifically select the BERT base-uncased model provided in the HuggingFace library [21], which returns a 768-dimensional vector as the class token for each text sample given as input.

We first pass the concatenated titles and abstracts of all surveyed articles to the BERT model in order to obtain the vectorial representation for each article. A limitation of BERT is that it accepts at most 512 text tokens (words) as input, which means that longer abstracts need to be trimmed at the end to fit this requirement. Out of the 84 surveyed articles, only 8 of them have the input text (composed of the concatenated title and abstract) longer than 512 tokens. This means that less than 10% of the articles are affected by this limitation. Even so, 7 of the overlength articles have less than 100 extra tokens. There is one article, that of Fisher et al. [3], with 226 tokens over the limit. In summary, the input length limitation of BERT does not have a significant influence on our clustering. Next, we employ the agglomerative clustering algorithm on the resulting vectors to obtain our AI-based hierarchy of articles, which can be visually presented as a dendrogram. The employed clustering algorithm is formally presented in Algorithm 1. The algorithm takes the set of articles and the pre-trained BERT model as input. It returns the dendrogram represented as a set of triplets. Each triplet in the returned set is composed of two clusters and the distance at which the respective clusters are merged. In steps 1 to 9, the algorithm computes the feature vectors for the given set of articles. In steps 10 to 12, the algorithm makes the proper initializations for the hierarchical clustering algorithm. In steps 13 to 18, the algorithm computes the increase in variance for every pair of clusters in the current set of clusters based on the Ward criterion. The variance of a cluster C is computed as follows:

$$Var\left(C\right)=\frac{1}{\left|C\right|}\sum_{v\in C}1-\frac{\langle v,\mu_{C}\rangle }{\left\|v\right\|\cdot \left\|\mu_{C}\right\|},$$

where $\mu_{C}$ is the mean vector of cluster C , |C| is the cardinal of cluster C , $\langle ∎,∎\rangle$ represents the dot product, and $\left\|∎\right\|$ is the $l_{2}$ norm of a vector. Note that the above formula employs the cosine similarity between each feature vector v and the cluster centroid $\mu_{C}$ . At step 19, the algorithm finds the pair of clusters with minimum increase in variance due to the merging. In steps 20 to 22, the clusters found are replaced by the clusters generated by merging the individual clusters. At step 23, the merged clusters, along with the distance at which they were merged, are stored in the solution Θ . The algorithm halts execution when all clusters have been merged into one big cluster. Note that the proposed clustering algorithm does not require any hyperparameters. We illustrate the resulting dendrogram and discuss our findings in Section 3.3.
**Algorithm 1.** Hierarchical clustering based on Ward linkage**Input:** 
$X=\{x_{1},x_{2},\ldots ,x_{n}\}$
—the database of articles, where each 
$x_{i}=(t_{i},a_{i})$
 is a tuple containing the title 
$t_{i}$
 and abstract 
$a_{i}$
 of the 
i
-th article in the database;

f
—the BERT pre-trained large language model.**Output:** 
$\Theta =\left\{\left(C_{i},C_{j},{∆}_{C_{i},C_{j}}\right)|C_{i},C_{j}\;\mathrm{are}\;\mathrm{two}\;\mathrm{clusters}\;\mathrm{merged}\;\mathrm{at}\;\mathrm{distance}\;{∆}_{C_{i},C_{j}}\right\}$
—the dendrogram represented as a set of triplets generated by the hierarchical clustering algorithm.**Computation:**1. 
V←∅;
 % Initialize the set of feature vectors with the empty set.2. for 
$x_{i}\in X$
 do3.  
$s\leftarrow t_{i}+\prime {\;}^{\prime }+a_{i};$
 % Concatenate the title and abstract of the 
i
-th article4.  if 
len(s)>512
 then % If the text has more than 512 text tokens (words).5.    
$s\leftarrow s\left[:512\right];$
 % Trim 
s
 to the maximum number of tokens accepted by BERT.6.  endif 7.  
$v_{i}\leftarrow f\left(s\right);$
 % Apply BERT to 
s
 and store the resulting [CLS] token to 
$v_{i}$
.8.  
$V\leftarrow V\cup \{v_{i}\};$
 % Add the computed feature vector to the set 
V
.9. endfor10. 
$C_{i}\leftarrow \left\{v_{i}\right\},\forall i\in \left\{1,2,\ldots ,n\right\};$
 % Initialize the leaf clusters with one sample per cluster.11. 
$\Psi \leftarrow \{C_{1},C_{2},\ldots ,C_{n}\}$
; % Initialize the set of clusters.12. 
Θ←∅
; % Initialize the dendrogram with the empty set.13. for 
k∈{1,2,…,n−1}
 do14.  for 
$C_{i}\in \Psi$
 do15.    for 
$C_{j}\in \Psi \backslash \{C_{i}\}$
 do16.      
${∆}_{C_{i},C_{j}}=Var\left(C_{i}\cup C_{j}\right)-Var\left(C_{i}\right)-Var\left(C_{j}\right);$
 % Compute Ward criterion.17.    endfor18.  endfor19.  
$A,B\leftarrow \underset{A,B\in \Psi ,A\neq B}{\mathrm{argmin}}\left\{{∆}_{A,B}\right\};$
 % Find the clusters with the smallest increase in variance.20.  
$C_{n+k}\leftarrow A\cup B;$
 % Merge clusters 
A
 and 
B
 into a new cluster denoted as 
$C_{n+k}$
.21.  
Ψ←Ψ\{A,B}
; % Remove clusters 
A
 and 
B
 from the set of clusters.22.  
$\Psi \leftarrow \Psi \cup C_{n+k};$
 % Add new cluster to the set of clusters.23.  
$\Theta \leftarrow \Theta \cup \left\{\left(A,B,{∆}_{A,B}\right)\right\};$
 % Store distance at which clusters 
A
 and 
B
 are merged.24. endfor

## 3. Results

### 3.1. Multiple Organ Failure in Oncologic and Non-Oncologic Patients

Subclinical multiple organ failure syndrome is a term used to define a subtle state or discrete symptoms associated with one or more organ dysfunctions in the absence of any clear evidence of organ failure. The present term can be used to describe a status in which several functional or biochemical alterations are present in an organ but are not evident enough to be diagnosed as clinical organ failure. Failure of an organ is described as the incapacity of an organ to fulfill its normal function; cardiac failure, renal failure, hepatic failure, pulmonary failure, and nervous system failure are several diseases that can lead to any organ failure and will be extensively discussed in this current review. In subclinical multiple organ failure syndrome, suboptimal functioning may exist but not be prominent enough to be diagnosed as a clinical syndrome. Another explanation might be that the suboptimal functioning of an organ cannot be observed due to compensation of function by the other organs, which seize its function in order to maintain the body’s homeostasis.

**Figure 1:**
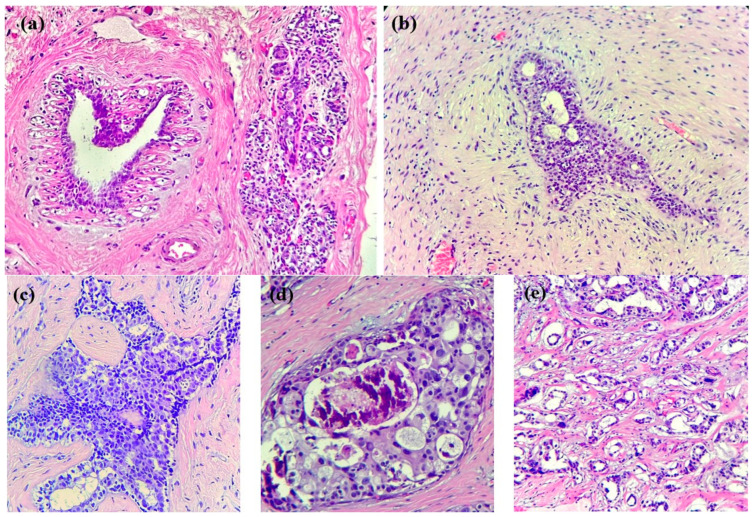
Breast tissue progression towards invasive cancer. (**a**) Normal breast tissue: ducts and lobules in a fibrous stroma, along with adipose tissue. (**b**) HE × 200. Usual ductal hyperplasia: benign intraductal epithelial proliferation with mild variation in cellular and nuclear size/shape. (**c**) HE × 200. Atypical ductal hyperplasia: intraductal clonal epithelial cell proliferation (<2 mm) with a cribriform pattern. (**d**) HE × 200. Ductal carcinoma in situ with high-grade atypia and cribriform growth pattern, comedonecrosis, and microcalcifications. (**e**) HE × 200. Invasive breast cancer of no special type (NST): infiltrative small nests and tubules of tumoral cells into a desmoplastic stroma.

In the case of breast cancer, its precursor lesions present specific morphological changes that precede cancer development (see Figure 1): typical ductal hyperplasia, atypical lobular hyperplasia, atypical ductal hyperplasia, in situ lobular carcinoma, and in situ ductal carcinoma represent some examples of these changes. These lesions are characterized by an abnormal growth of ductal/lobular mammal cells, which are not yet in a malign state but have a higher capacity than normal cells. In our era, these lesions might be incidentally discovered on minor ultrasound and mammography [22] and subsequently diagnosed via histopathological examinations. It is mandatory to note, however, that not all lesions will progress into invasive cancer; the occurrence of external factors is the prerequisite for such an evolution.

**Figure 2:**
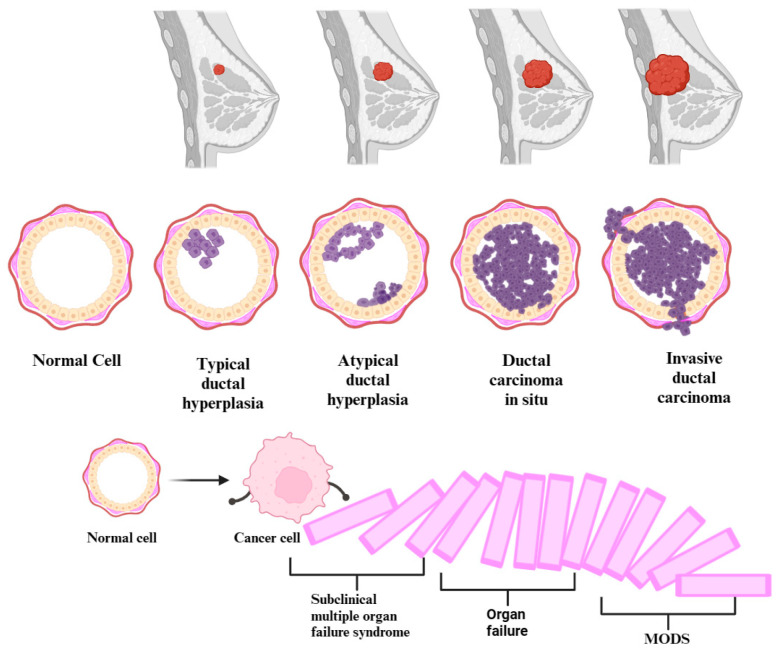
Development of multiple organ dysfunction syndrome (MODS) from subclinical multiple organ failure syndrome in the context of the natural prognosis of breast cancer.

The precursor lesions of breast cancer (typical ductal hyperplasia, atypical ductal/lobular hyperplasia) and the subclinical syndrome of multiple organ failure constitute early stages in certain diseases. These stages are characterized by alterations that manifest before apparent symptoms become clinically evident or identifiable through conventional diagnostic methods. Equally, cancer and multiple organ dysfunction syndrome (MODS) are progressive diseases. In the beginning, the homeostasis of the body is altered by infectious and non-infectious factors. Thus, the occurrence of changes in pathophysiology, which can start from the genetic level and up to the clinical manifestation, makes MODS evolve from a subclinical syndrome of organ failure, through the domino effect, up to an acute, clinical, multiple organ failure, resulting in severe disease. Breast cancer gradually progresses through genetic and molecular subclinical alterations, transitioning from the initial, usual ductal hyperplasia stage to advanced metastatic breast cancer (see Figure 2). In certain conditions, it represents a form of organ failure, depending on where it metastasized. Both conditions usually evolve in stages, going through a progressive process of deterioration. Therefore, given the similarities in the genesis of the two diseases, we can posit a paradigm shift. This shift is directed towards the fact that cancer precursor lesions, those with the potential to evolve into cancer, might independently initiate a subclinical multiple organ failure syndrome through their presence. In the context of breast cancer, the metaphorical cascade of dominoes undergoes a complete upheaval during the metastatic stage. A single organ failure occurs when the disease progresses uncontrollably, extending to a specific organ. Subsequently, this initiates a cascade effect, triggering additional insufficiencies and culminating in the manifestation of multiple organ failure syndrome.

Furthermore, it is crucial to note that inflammation and altered immune responses are prevalent in both conditions. Specifically, in the context of MODS, inflammation and compromised immune responses may contribute to organ dysfunction associated with cancer. Alternatively, the interplay of chronic inflammation and the body’s immune response is recognized to exert influence on tumor development and progression, thereby impacting cellular and tissue functionality. Both pathologies show organ failure. MODS is characterized by dysfunction and failure of at least two or more organs, while cancer, in advanced stages, can affect the functioning of one, two, or more vital organs, depending on the location and extent of the tumor. Thus, the processes associated with the growth and progression of cancer, in the absence of treatment, over time, will determine the onset of MODS. Starting from MODS, which is known in the literature and has been studied for several decades, we make a retrospective analysis of its evolution processes, trying to describe or observe the subclinical multiple organ failure syndrome, the starting point of an organ failure. The difference between these two conditions lies in their severity and symptom manifestation. The subclinical multiple organ failure syndrome is a more subtle and less obvious form of multiple organ failure, with the potential for reversibility being compensated by the body through its own homeostasis. Thus, its symptoms are not obvious enough to be detected by regular clinical tests. Quantifying this syndrome proves challenging due to the absence of manifested symptoms, leading to its discreet progression through distinct stages until reaching the state of MODS. In parallel to the progressive diagnosis and treatment of cancer, an ideal approach would involve diagnosing and staging organ failure. This would enable the initiation of early therapeutic management, thereby averting the progression of the body towards MODS.

Therefore, the diagnosis of subclinical multiple organ failure syndrome may demand extensive utilization of laboratory examinations, medical inquiries, imaging techniques, and advanced innovative instruments, such as artificial intelligence (AI), to detect those subtle alterations linked to organ dysfunction before the patient exhibits any visible symptoms and, through the domino effect, develops MODS.

Multiple organ dysfunction syndrome (MODS) or multiple organ failure (MOF) was first mentioned in 1991 by the Consensus Conference of the American College of Chest Physicians and the Society of Critical Care Medicine. It was defined in acutely ill patients as an organ dysfunction that needs intervention to maintain its homeostasis. MODS and MOF are still used interchangeably, but several other terms have become more prevalent: persistent, progressive, or secondary MODS. Actual data suggest that MODS is two times higher in patients with comorbidities. Thus, an increase in death by 60% is observed when correlated with the number of organ failures [23].

**Figure 3:**
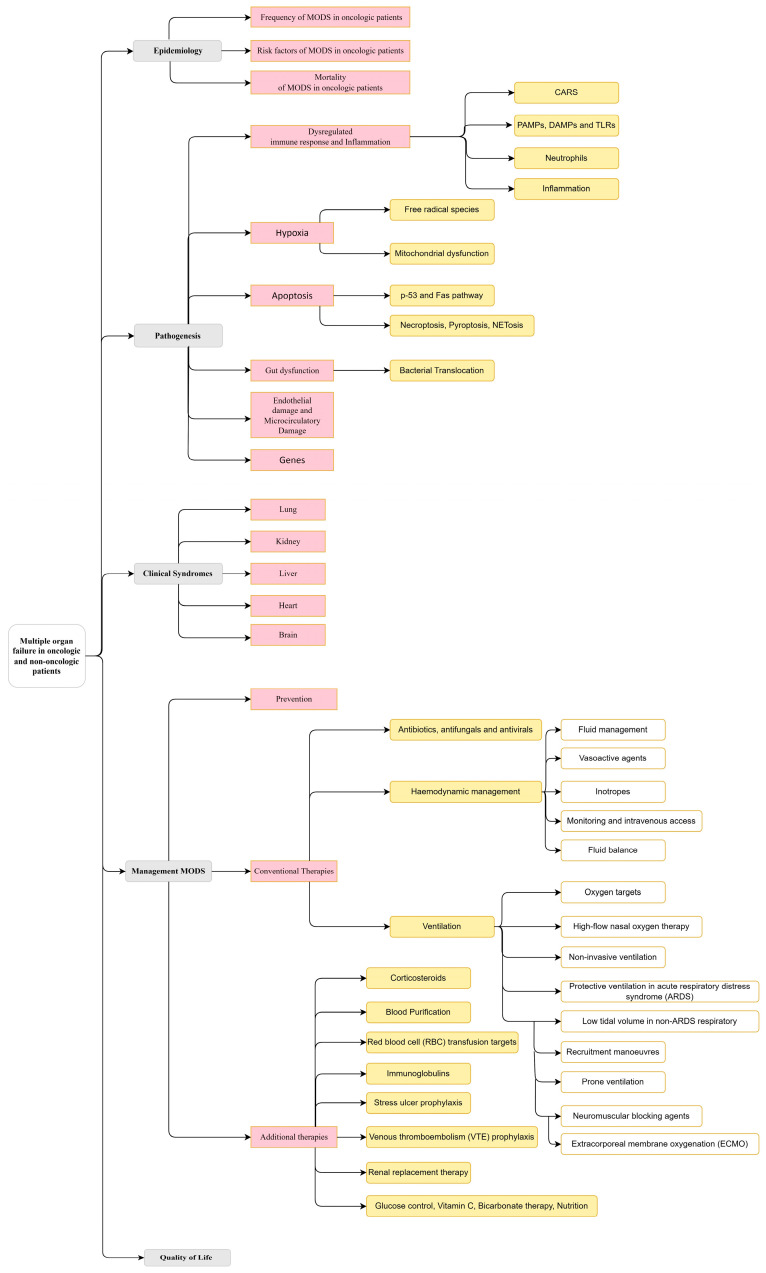
A taxonomy of the multiple organ dysfunction syndrome (MODS) in the actual context of oncological and non-oncological patients.

This review is structured according to Figure 3.

#### 3.1.1. Epidemiology

- Frequency of MODS in oncologic patients

The epidemiology of the MODS in breast cancer patients can vary according to numerous factors: stage of disease, complications of breast cancer, used therapies, and individual particularities. Initially, infections were considered the primary source of MODS. In addition, MODS as a result of sepsis was observed to increase from 19.1% to 33.6% in the last subperiod [24]. A study of the epidemiology of severe sepsis in the USA showed that the most common site of infection was the respiratory tract, which correlated with mortality: 21.2% for one organ failure, 44.3% with two organ failures, 64.5% with three organ failures, and 76.2% with four or more organ failures [25]. Another study showed that 50% of ICU admissions were due to sepsis, with respiratory failure associated with mortality and some of the risk factors being cancer-related [26]. In addition, mortality was correlated with other comorbidities, such as chronic lung disease, kidney disease, liver disease, complicated diabetes, and malignancy [25].

Oncologic patients face a tenfold higher susceptibility to sepsis compared to non-oncological patients. Moreover, they are more prone to ICU admission attributed to sepsis, with recent studies reporting mortality rates ranging from 40% to 60% [27]. However, since 2016, sepsis has been defined as Sepsis-3, and it represents a multiple-organ dysfunction syndrome caused by an irregular response of a host to infection. Therefore, what mediates MODS could explain the pathophysiology of sepsis [28]. Regarding oncologic patients, they are at significant risk of reaching a critical phase of disease. This observation was made in a study encompassing prevalent malignancies such as lung cancer, colorectal cancer, and breast cancer, with breast cancer being notably associated with the lowest risk of entering a critical illness state [29]. Moreover, the diagnosis of cancer on admission to an intensive care unit was variably common, with rates ranging from 13.5% to 21.5%, with an outcome dependent on the type of admission [30]. Breast cancer was the most frequent malignancy (20.2%) in the primary population of a study, with reasons for admission represented by sepsis and infection (18.5%) and surgery for cancer (23.6%) [30]. In concordance with the previous study, some data suggest that breast cancer patients represented the majority of studied participants (14.4%), with infection or sepsis being the most common reason for admission (31%) [31]. In addition, in another study, ICU mortality in oncologic patients was 74% when first presented due to complications related to cancer treatment, with causes of death (48%) being from refractory shock or MODS [32]. Correspondingly, 66% of cancer patients were newly diagnosed when they first arrived in the ICU; sepsis and multiple organ failure were the most common causes (32%) of admission [33].

Although breast cancer incidence is elevated in female populations, data are scarce when put in the context of intensive care and multiple organ failure. Some data show that 42.4% of breast cancer patients were admitted mainly due to infection (31.6%), leukopenia (8.4%), thrombo-embolic events (3.4%), and neurological complications (2.8%) [34].

- Risk factors of MODS in oncologic patients

Risk factors that could lead to the onset of multiple organ failure syndrome in breast cancer patients are an advanced stage of disease, aggressive treatments, complications of breast cancer, secondary reactions after therapy, and the presence of comorbidities. Mizock et al. emphasize that hypoperfusion and sepsis with or without shock, or purely shock, irrespective of etiology, are the most common risk factors [35,36]. Aagaard et al. describe febrile neutropenia (FN) as frequently associated with not only breast cancer but also with ICU admission and organ failure. They observed that FN was a risk factor that increased the non-specific cause of mortality in cancer patients by 38% due to a two-fold increased risk of mortality by infection [37]. Moreover, chemotherapy was also found to be a factor that leads oncologic patients to develop FN, followed by shock or severe sepsis, and arrive in ICU settings, with rates that range from 7.9% to 11.7% [26,37]. Brass et al. describe oncologic risk factors such as surgery, radiotherapy, chemotherapy, bone marrow transplantation, and biotherapy to be used with care. Surgery, being one of the most utilized forms of cancer treatment, promotes thrombosis and dysfunctional coagulation, which can lead to an irregular activation of the immune system. Radiation therapy and chemotherapy, during their effects on tumoral cells, also damage the healthy normal cells of the body, which could finally lead to renal and liver damage, promoting abnormal coagulation activation and a dysfunctional immune response. Moreover, bone marrow transplantation and chemotherapy or radiation therapy have multiple powerful effects on the systems of the body, which ultimately suppress the immune response. Lastly, the development of biotherapy can suppress the immune system with direct effects on the inappropriate immune response [38]. Data showed the risk of being admitted to the ICU was surgery alone (11.8%), surgery and both chemotherapy and radiotherapy (10.5%), chemotherapy plus surgery (9.3%), and radiation plus surgery (8.1%) [30]. McFadden et al. reinforce that the therapeutic mechanism involved in cancer treatment could lead to complications such as infection, fever, gastrointestinal bleeding, multiple transfusions, splenectomy, and intra-abdominal infections, which could finally promote the onset of MODS [1].

Other associative risk factors are debated in the commencement of MODS. Salluh et al. exposed antiphospholipid antibodies (aPL) in cancer patients and their association with high rates of thrombosis and an unfavorable outcome [39]. Respiratory failure and acute respiratory distress syndrome (ARDS) in cancer patients have several causes: infections, direct hits on the respiratory system, cancer-related disorders, and oncologic therapy. In addition, in neutropenic oncologic patients, ARDS exhibits exclusive features, and caution should be taken [40]. Inadequate, delayed resuscitation, prolonged infection, persistent inflammatory state, renal insufficiency, age over 65 years, excessive alcohol use, malnutrition, surgical operations, diabetes, steroids, and cancer [41], as well as thrombocytopenia, were additional risk factors implied in the development of MODS [42].

- Mortality of MODS in oncologic patients

As previously mentioned, mortality increases as the number of failing organs increases, as well as the number of days in the ICU. Three organ failures lasting for more than three days were associated with a 71.4% death rate [27]. The reported death rates in solid tumors vary in the literature, ranging from 50% [43] (36.6% in breast cancer [26]) to 90% [44], while for non-cancer patients, the death rate represents nearly 20% [43]. Similarly, in a study with 20,000 cancer patients, those with newly diagnosed malignancy were at a 4.6 times higher risk of mortality in the first year after being discharged [30]. Data that studied patients with breast cancer and their admission to the critical care unit showed that two or more sites of metastasis had a reduced chance of survival. In addition, a reserved prognosis was observed in patients with a progressive or unresponsive status of malignancy or granulocytopenia [44,45]. Moreover, the mortality in neutropenic cancer patients with respiratory failure and cardiovascular failure was 81%, while in those with neurological failure, the death rate was 80%, and with renal failure, 65% were deceased. Thus, due to the development of aggressive myelosuppressive chemotherapy for solid cancers, long and harsh neutropenia could be observed for any given malignancy [44].

In contrast to earlier findings, however, no evidence of the type of cancer was detected to influence mortality in ICU settings [44]. It is worth mentioning that patients who survived the MODS event have a reduced long-term quality of life in physical, cognitive, and psychological domains, with an essential impact on health care and the social environment [46].

#### 3.1.2. Pathogenesis

Several theories are trying to explain how multiple organ failure develops. However, due to its numerous molecular mechanisms and genetic involvement, the pathophysiology of MODS remains stimulating and still to be explored [47].

Two decades ago, Marshall et al. described MODS by defining the concepts of sepsis and systemic inflammatory response syndrome (SIRS) [48]. They proposed that the host’s response is the most important element of the outcome, instead of the cause that promoted the onset of MODS. Five pathological mechanisms were advocated: uncontrolled infection, dysregulated immune response, hypoxia of the tissue, uncontrolled apoptosis, and microvascular coagulopathy. Moreover, Ader et al. introduced the notion of psychoneuroimmunology, in which MODS can result due to disturbance of the immunoinflammatory, endocrine, and nervous systems, as well as behavioral and emotional status [49].

Multiple factors are thought to be generators of MODS’s pathophysiology. The immunoinflammatory system is considered to play a key role. The gravity of organ dysfunction and its sequence in patients with MODS can be emphasized using cytokine storms, adhesion molecules, and intrinsic inflammatory cells [50]. However, the present knowledge of the pathogenesis of MODS points to a variety of cell populations, metabolites, hormonal systems, and neural signals, in addition to oxygen delivery and utilization impairments and cell phenotype changes. Moreover, cell or tissue hypoxia, induction of apoptosis, gut microbial translocation, immune dysregulation, and mitochondrial impairments are intensively studied [51].

Recently, more models to explain the commencement of MODS have been intended. The “one-hit” model suggests that organ failure develops as a result of massive direct damage. The “two-hit” model describes an initial insult followed by a second hit, such as a catheter infection, which maintains the inflammation and the immune dysfunction. The third model is a “sustained-hit model”; a prolonged insult occurs, such as in ventilator-associated pneumonia (VAP), which causes the initial damage and the sustained dysfunction [52].

Emerging evidence, while others are still to be found, intends to point out that immune system dysregulation is linked to an uncontrolled immune response that generates free radical species and cellular and tissular hypoxia that sustains the production of free radical metabolites. Therefore, mitochondria’s function is damaged, which is thought to be a prevailing mechanism in MODS’s pathogenesis. In the pages that follow, current theories regarding MODS pathophysiology will be briefly discussed.

- Dysregulated immune response and inflammation

A dysregulated immune response is characterized by an imbalance between pro-inflammatory and anti-inflammatory factors. As a defensive mechanism against pro-inflammatory cytokines, anti-inflammatory cytokines such as interleukin-10 (IL-10) are generated to sustain the body’s homeostasis. This process is known as compensatory anti-inflammatory response syndrome (CARS). The main role of CARS is to restrict the damage done by pro-inflammatory factors while permitting pathogen destruction. However, CARS can lead to an unwanted phenomenon such as “immunoparalysis”, a dysregulated immune response, which further leads to injury and infection due to the host’s vulnerable status [51].

Damage-associated molecular patterns (DAMPs) and pathogen-associated molecular patterns (PAMPs) are molecules specifically designed to respond rapidly to injury or infection to maintain the body’s homeostasis. PAMPs are exclusively microbial molecules that, once identified, alert the human organism, while DAMPs are similar but are produced solely as a consequence of endogenous damage [53]. Toll-like receptors (TLRs) are the receptors through which DAMPs and PAMPs initiate the inflammatory innate immune response [54]. After activation, immune cells, predominantly macrophages, produce proinflammatory cytokines such as tumor necrosis factor-alpha (TNF-α) and interleukin-1 beta (IL-1β). These cytokines promote further production of other pro-inflammatory molecules, and more leukocytes are recruited. Finally, other inflammatory molecules, such as high mobility group box 1 protein (HMGB-1) and interleukin-6 (IL-6), are secreted, which will sustain the inflammation [55]. Moreover, DAMPs were associated with initiating the immune response, promoting coagulation, and sustaining MODS. Therefore, DAMPs, PAMPs, and TLRs are key factors in the appearance and progression of sepsis [28].

As to neutrophils, they also play a significant role in the dysregulation of the innate immune system. Cytokines such as TNF-α induce neutrophil alteration of surface protein synthesis, adhesions with vascular endothelium, dissemination in extravascular sites, and synthesis of superoxides. During inflammation, neutrophils enter an apoptotic downregulation state, giving rise to relative “immortal” neutrophils [51]. This process of self-perpetuating neutrophils and macrophages is not yet completely understood. Following the exaggerated self-perpetuating process, various modifications in mitochondrial, endothelial, epithelial, microcirculatory, coagulation, and neuroendocrine functions emerge. These alterations are likely attributed to genetic, molecular, cellular, and mediator modifications [2]. Therefore, a substantial influx of neutrophils occurs in organs enduring organ failure. The impairment of one organ has the potential to instigate the recruitment and dissemination of neutrophils in other organs, ultimately culminating in multiple organ failure. These observations promote the idea that neutrophil dysfunction is a consequence of systemic activation and not a primary mechanism in severe sepsis [55].

Currently, inflammation is the most accepted paradigm as an etiological explanation of MODS. Inflammation is characterized by the activation of leukocytes, endothelium, and multiple mediators that, in normal conditions, are held in balance due to anti-inflammatory mediators. Therefore, MODS occurs when either a pro-inflammatory or anti-inflammatory response to damage is excessive. However, death may result if the response is either too excessive or too inefficient. A variety of pro-inflammatory molecules are released by macrophages: TNF, IL-1, and IL-6. After synthesis, these molecules upregulate the receptors of neutrophils such as L-Selectin, and the endothelial receptors such as P-Selectin, E-Selectin, Intercellular Adhesion Molecule 1, and Vascular Cell Adhesion Molecule 1 Cellular [56]. Therefore, numerous physicians use cytokines as a prognostic marker in patients with SIRS, sepsis, or MODS. Regarding this context, a value of 800 pg/mL of IL-6 is proposed as a threshold level that can aid in differentiating patients with or without organ failure [57]. In addition, levels of IL-6 were correlated to early tissue development and more predictive for MODS onset than injury severity, age, or sex in some patients [58]. Interleukin-8 (IL-8) and TNF-α are well stimulated in MODS, and the extensive involvement of TNF-α determined scientists to find therapies against it [59]. However, some studies of anti-TNF-α therapies did not show a significant increase in survival rate in sepsis patients [60]. Therefore, the relationship between inflammatory activity and immunosuppression in critically ill patients persists as a great challenge in modern intensive care units [2].

- Hypoxia

Systemic cellular and tissue hypoxia was discussed as a precipitating factor in organ dysfunction. However, in sepsis patients, these changes in oxygen levels are more subtle. Oxygen use becomes dependent not only on oxygen delivery but also on cellular processes. Thus, an elevated oxygen gradient must be obtained in mitochondria. Increasing oxygen levels also improves oxygen extraction, through which morbidity and mortality are reduced [47]. An additional issue is represented by the formation of free-radical species. Reactive oxygen species (ROS) conduct the generation of reactive nitrogen species (RONS) and ferric species, resulting in direct cellular damage and a secondary inflammatory cascade. While RONS are produced by immune cells and delivered into extracellular space to destroy pathogens, they can additionally damage the host cells due to uncontrollable activation. Moreover, ROS and RONS are released within the cells, and their balance could induce the progression of organ failure. Phagocytic cells then use ROS to eliminate the pathogens. However, the action of ROS may become quickly dangerous to the host’s body due to lipid peroxidation and protein degradation [61]. Therefore, an association between ROS discharge, organ dysfunction, and clinical outcome has been made [62].

In the systemic inflammatory response, mitochondria—the foremost producer of ROS—also generate elevated levels of RONS through the RONS-NOS cycle [61,63]. Therefore, ROS, RONS, and carbon monoxide may damage the mitochondrial membrane, impair key mitochondrial enzymes, and reduce mitochondrial respiration, ending with mitochondrial apoptosis. Crucially, reduced oxygen levels decrease ATP production, promoting necrotic cell death. Directly involved or not, compromised mitochondrial function promotes “cytopathic hypoxia” [63], a paradigm that supports the disconnection between optimal oxygen delivery and poor oxygen utilization at the tissular level in sepsis-induced MODS [51]. Thus, in experimental bacterial sepsis, pharmacologic reduction in mitochondrial impairments could represent a therapeutic target due to its status as a factor that can cause MODS [64]. Antioxidant therapies also reduce inflammation and MODS development. This sustains the concept that pro-inflammatory molecules disrupt the plasma membrane, while hypoxia impairs both intracellular structures and the plasma membrane [61]. Moreover, the actual findings point out that DNA-dependent protein kinase catalytic subunit (DNA-PKcs) expression induces mitochondrial damage in the heart, kidney, and liver in MODS due to sepsis. Nonetheless, the alterations of the mitochondrial regulatory pathway are not yet completely studied, even if mitochondrial dysfunction is a well-known early marker of sepsis-induced MODS [65]. In addition, whether mitochondrial damage initiates organ failure or results as a consequence of the inflammatory process in infection, the precise mechanisms of how mitochondrial impairments both determine and are determined by critical illnesses remain to be discovered [63].

- Apoptosis

Programmed cell death, or apoptosis, is a main finding in MODS. Firstly, cell death represents an adaptative response to limit tissue necrosis. However, an overreaction may not be beneficial to the organism [63]. It is accepted that high mortality rates and organ dysfunction in sepsis patients are due to the massive apoptosis of immune cells [66]. Excessive apoptosis of T and B lymphocytes could weaken the immune response against pathogens [63]. However, no direct linkage between immune cell death and immunosuppression has been made [61]. Mitochondrial pathways and death receptors are activated by multiple factors, which will induce apoptosis in a large population of lymphocytes. In addition, apoptosis could follow p-53-dependent and independent pathways. Overexpression of B-cell lymphoma 2 (BcL-2), an anti-apoptotic protein, has been observed following decreased cell death in gut epithelia and has been associated with better survival rates in sepsis mice. Moreover, the death of Fas receptors has been involved in the pathogenesis of acute lung injury and ARDS due to sepsis and in high levels of mortality in ARDS patients [63].

Recently, other processes such as necroptosis, pyroptosis, ferroptosis, parthanatos, entotic cell death, neutrophil extracellular traps (NETs) cell death, immunogenic cell death, and mitotic catastrophe have become intensively studied [67]. Necroptosis was attributed to the death of immune cells due to its activation by TNF-α in critical care illnesses. Elevated permeabilization of the membrane in necroptosis permits the release of DAMPs, while lipid peroxidation happens in ferroptosis and may occur in renal dysfunction. Ferroptosis was also associated more recently with vascular leakage in septic conditions. Pyroptosis is caused by rapid plasma membrane disruption due to the non-selective gasdermin-D pore, which permits DAMP excretion. Therefore, pyroptosis of neutrophils and endothelial cells is considered a major factor in the pathogenesis of sepsis. NETosis has also been reported as being involved in the onset of MODS in sepsis [61]. Abrams et al. recently observed that the powerful NET formation that occurs in severe sepsis promotes disseminated intravascular coagulation (DIC) and induces a poor outcome [68]. These NETs are degraded into histones and free DNA, becoming sources of DAMPs. Extracellular histones fix into the cell membrane, create pores, and permit the influx of calcium, ending with cell death [28]. As a consequence, neutralizing antibodies, heparins, and C1 esterase inhibitors (C1-INH) were used as anti-histone reagents and were able to reduce histone toxicity. However, more data are essential to observe whether or not circulating histone has a significant role in secondary hits in MODS [69].

Preventing NETosis in oncologic patients could be used as a strategy to prevent thrombosis induced by tumors and suppress metastasis and organ failure. A lot of factors are acknowledged in lymphocyte and neutrophil apoptosis, but more specific molecular mechanisms are needed. Therefore, new clinical trials of therapies in MODS for sepsis emerged [70].

- Gut dysfunction.

The gastrointestinal (GI) system is formed by a unicellular epithelial layer and both a local immune system and a microbial environment. These structures have long been speculated to be the engine of MODS due to multiple organ failure syndrome’s capacity to disrupt the body’s homeostasis. As a result, a shift in the beneficial microbiome occurs, accompanied by an uncontrolled immune response. During this process, the intestinal barrier experiences increased permeability, allowing pathogens to enter extraluminal spaces and adjacent lymph nodes. More hypotheses about the role of the gut in MODS have been developed. The first one proposes that bacterial extravasation is promoter of MODS. Critical diseases induce changes in the nature and quality of mucus layers. Inflammation, hypoperfusion at the gut level, and ischemia–reperfusion reactions are considered potential causative factors. As a result, bacteria may traverse through the intestinal barrier, reaching mesenteric lymph nodes (MLNs). Concomitantly, endotoxin liberation is believed to promote the systemic inflammation that leads to MODS. However, only the indirect linkage between bacteria and endotoxin translocation is observed in critically ill patients with sepsis and MODS. The second one proposes a link between MODS after a critical insult and the discharge of non-bacterial pro-inflammatory mediators from a “stressed” intestine. Injury to the tissue begins when these molecules arrive in the systemic circulation via the mesenteric lymphatic system. At that level, MLNs have no bacteria and even low levels of endotoxins or cytokines. However, MLNs present high levels of protein and lipid molecules that act as “danger molecules”, further stimulating TLR-4 [47].

Clinical data provided by Shimizu et al. showed that synbiotics, such as Bifidobacterium breve strain Yakult and Lactobacillus casei strain Shirota, significantly reduced enteritis and VAP. Moreover, probiotics could provide security in ICU settings against intestinal inflammation or even postoperative sepsis [71].

- Endothelial Damage and Microcirculatory Damage

Coagulopathy and disseminated intravascular coagulation (DIC) in the context of sepsis play a significant role in MODS development [28]. Virtually, regulation of vascular tone, barrier role, inflammatory capacity, and all roles of the endothelium are involved in MODS. The luminal layer of endothelial cells enters a prothrombotic state, which promotes the onset of DIC. In addition, this layer becomes more permeable, followed by fluid extravasation and edema. Activated endothelial cells release nitric oxide, which leads to vasodilation, hypotension, and septic shock, while neutrophils adhere to the surface molecules and stimulate inflammation. Therefore, the peeling of endothelial glycocalyx uncovers other hidden adhesion molecules that aid the fixation and transmigration of leukocytes into the parenchyma. As a consequence, capillary leakage, inflammation, platelet aggregation [72,73], coagulation, and the loss of vascular tone occur [63]. Sepsis and oncologic patients usually have a degree of coagulopathy, which is manifested through thrombocytopenia and DIC in advanced stages. These disturbances result from the activation of the coagulation pathway, the inhibition of the anticoagulation pathway, and the diminished function of the fibrinolytic mechanism [74].

- Genes

Several genes are studied as potential factors involved in the development and outcome of MODS. Gourd et al. focused their attention on proteins, such as the Janus kinase family and mitogen-activated protein kinase (MAPK), and genes involved in the p53 pathway, IL-6, TLR, and plasminogen activator inhibitor 1 (PAI-1). Pro-inflammatory cytokines promote the activation of the Janus kinase transducer and activator of the transcription pathway (JAK-STAT). MAPK is a serine/threonine protein kinase with multiple functions in cellular processes such as growth, proliferation, stress response, and apoptosis. In the context of MODS, MAPK pathways were studied in the pathogenesis of multiple organ failure in hemorrhagic shock and lung injury induced by ventilation. For example, in mouse studies, inhibition of MAPK reduced the quantity of infiltration in lungs and intestines with polymorphonucleocytes (PMN), the production of pro-inflammatory cytokines, and the amplification of intracellular adhesion molecules. This reduces tissue damage as a result of decreased apoptosis. Growth arrest and DNA-damage-inducible protein alpha (GAD45A), a protein coded by the p53 gene, have been studied in MODS commencement in mice with lung injury, in which their values were elevated. However, this theory needs further studies to be validated. Polymorphisms in genes implied in IL-6 and TLR synthesis were examined as a factor that increases the risk of septic complications and MODS development in children patients. In mouse models, different gene expressions involved in TLRs, TNF receptors, epidermal growth factor (EGF) signaling, and nuclear factor kappa B (NF-kB) have been studied with mRNA analysis. Evidence showed an interaction between an EGF receptor node, transcription factor jun-B (JUNB), STAT3 transcription activator, and IL-6. In addition, in patients with pneumonia and both MODS and septic shock, some polymorphisms in PAI-1 were observed [2].

#### 3.1.3. Clinical Syndrome

Temporal evolution and clinical manifestations in multiple organ failure syndrome are influenced by several individual factors (advanced age, comorbidities, immunosuppressive therapies) [75] and genetic factors [35]. Sauaia et al. emphasized that while the incidence of lung failure decreased, it sustained its status as the primary organ failure throughout the study period. In contrast, cardiovascular failure exhibited a reduction, and liver and renal dysfunctions remained relatively stable at basal levels. Moreover, a decrease in evolution from lung failure to multiple organ failure dysfunction (MODS) was also observed. MODS without lung failure is rare, and only 8% of patients did not present a lung association [76].

- Respiratory

The pulmonary system is commonly the first system damaged in MODS. Usually, MODS patients present with ARDS. Its incidence varies with different risk factors and is estimated to be present in 1.5 to 5.3 cases per 100,000 people [45]. Several illnesses have been linked to ARDS in cancer patients. However, it is difficult to assess the risk of developing ARDS due to a subtle insult. Acute respiratory distress syndrome (ARDS) is the main phenomenon of pulmonary dysfunction in humans and animals. Two different pathways are proposed to be the cause of ARDS: direct pulmonary damage (bacterial or aspiration pneumonia, inhalation injury, lung trauma) and indirect pulmonary damage (sepsis, burns, blood transfusions, pancreatitis) [51]. The pathogenesis of ARDS is characterized by the infiltration of neutrophils in the pulmonary parenchyma, alveolar-capillary barrier disruptions, pulmonary vascular leakage, and the alveolar and systemic secretion of proinflammatory cytokines. Pulmonary edema appears as a result of the alveolar-capillary barrier break and increased vascular permeability, while inflammation, atelectasis, and damage to type I alveolar pneumocytes result from pro-inflammatory cytokines. Moreover, for patients who died of ARDS, post-mortem biopsies showed the presence of an augmented number of platelets and neutrophils in pulmonary vessels [51]. In oncologic patients, the leading causes of respiratory failure are infections, the direct hit of the pulmonary system, cancer-related medical diseases, and antitumoral therapy drug-induced respiratory distress [77].

- Kidney

Acute kidney injury (AKI) is commonly referred to as renal dysfunction. Osterbur et al. emphasized that in MODS patients, two central forms of AKI can be observed. The first one implies renal epithelial necrosis, with renal hypoperfusion and ischemia as common pathogenesis. The second one is characteristic of MODS patients and is not attributed to necrosis. While the first one is the least present in the population, with only 22% of patients with AKI-induced sepsis, the second one is the most frequent, with apoptosis as the main pathogenesis due to pro-inflammatory cytokines such as TNF-α and endotoxins. Even if AKI is commonly associated with sepsis patients, its pathogenesis and management remain disputed. Leukocyte infiltration could be an explanation due to its presence in septic kidneys in animal models and humans, but leukocyte depletion also seemed to reduce renal damage. Moreover, recent studies have shown that renal failure continues to develop even if global renal blood flow is maintained in severe septic patients [51].

Renal dysfunction can be measured using two laboratory values: rate of glomerular filtration (RFG) and serum creatinine concentration. In oncologic patients, acute renal dysfunction is commonly due to multiple causes. Therefore, careful surveillance and management of renal function in malignancy are mandatory in order to maintain the effectiveness of the treatment and the outcome of the illness. Nevertheless, in the event of renal impairment, it becomes imperative to implement judicious adjustments in medication dosages. Additionally, medical interventions aimed at ameliorating renal function should be undertaken, accompanied by vigilant monitoring of kidney function. This proactive approach is essential in addressing and managing potential complications arising during oncological treatment.

- Liver

Liver function can be assessed using bilirubin concentrations. However, due to the extensive capacity of the hepatic function to process the bilirubin, an important hepatic injury can appear before the onset of the icterus [78]. In ICU settings, the common causes of hepatic dysfunction can be sepsis, ischemia or reperfusion injury, massive heme substrates such as exaggerated transfusions, and exclusive parenteral nutrition [36]. Even though circulatory shock can cause acute liver damage as a consequence of diminished hepatic perfusion, this phenomenon is short in duration and commonly disappears after a proper hemodynamic resuscitation. Therefore, a subclinical state is usually present, followed by liver dysfunction due to extensive hepatic inflammation [35].

Regarding liver dysfunction in the context of breast cancer, it can occur as a consequence of direct damage because of hepatic metastases or indirectly due to aggressive treatments for oncologic disease. The control of hepatic dysfunction in breast cancer patients may be addressed with symptom control, medication that alleviates hepatic function, metastasis excision, and dosage reduction to diminish the adverse effects.

- Cardiocirculatory system

In MODS patients, two central mechanisms are involved: hypoperfusion and myocardial dysfunction. Neutrophil interaction with endothelial cells, endothelial dysfunction, microvascular flow derangements, and intravascular coagulation during ischemia, whether accompanied by reperfusion injury or not, may contribute to the dysfunction of various organs. The organs affected include those previously mentioned (lung, kidney, liver), heart, and brain. In addition, heart function can be impaired by oxygen and nitrogen reactive species, pro-inflammatory cytokines such as TNF-α, and lipopolysaccharides [36].

Paraclinical findings in cardiovascular dysfunction, such as biventricular dilation, diminished ejection fraction, hypotension in spite of volemic replacement, and an abnormal response to catecholamines, may be observed. Thus, multiple biocellular substances associated with a decrease in myocardial contractility are involved in the onset of cardiovascular dysfunction. Endotoxin, cytokines such as IL-1β, TNF-α, platelet-activating factor, and calcium extravasation from the sarcoplasmic reticulum represent some of the mechanisms involved in this process. In sepsis patients, cardiovascular impairment is common, with some studies presenting it at up to 66%. Moreover, it is observed as a reserved prognostic factor, being associated with elevated mortality rates up to 70% [51].

In the septic process, peripheral circulation also plays a role. Vasodilation in peripheral vessels gradually progresses as MODS evolves into clinical organ failure and becomes refractory to vasoconstriction therapy. This path in sepsis is discussed as being mediated by the erythrocytes’ capacity to store and deliver nitrogen oxide in the state of S-nitrosothiols [35].

Coagulation disturbances or other coagulopathies are commonly present in both septic and oncologic patients, with some studies emphasizing their presence up to 80%. DIC is an illness caused by an uncontrollable systemic activation of the clotting cascade due to pro-inflammatory cytokines, such as IL-1β, IL-6, and TNF-α, followed by excessive consumption of clotting factors, microvascular thrombosis, and finally, thrombotic or hemorrhagic episodes [51,79]. DIC usually occurs in sepsis, cancer, and trauma [51]. However, no clear-cut therapy has been studied so far, and DIC management and its complications in acute sepsis states remain difficult. Due to these reasons and the ineffectiveness of specific anticoagulation treatments in reducing mortality in clinical trials [79], the mortality rate remains notably high. This heightened mortality is attributed to its association as an independent predictor of death. In addition, the number of platelets is utilized to quantify the dysfunction of the coagulation system [36]. However, this investigation has some impediments for oncologic patients who are currently under malignancy treatments because thrombocytopenia may follow due to adverse drug reactions.

In summary, cardiac failure in breast cancer patients may be due to a variety of factors. Comorbidities and several therapies, such as Trastuzumab, produce cardiac toxicity and, therefore, cardiac failure. Thus, periodic evaluations are performed during and after treatment on these patients for early detection of cardiac failure.

- Brain

The most frequent encephalopathy in the ICU setting is septic encephalopathy (SE) or sepsis-associated encephalopathy (SAE), with a prevalence ranging from 9% up to 71%, based on what criteria are defined. SE is suggested to be an alteration of the functional status without structural changes, although magnetic resonance imaging and cognition in the long term seem to show some levels of structural disturbances. However, its pathogenesis is not yet fully known. A lax blood–brain barrier due to increased permeability permits the entrance of aromatic AA-derived neuroamines that lead to an impairment in mental status. Nonetheless, other studies hypothesize that pro-inflammatory cytokines enter through the more permeable blood–brain barrier, therefore promoting the onset of SE [35]. Key pro-inflammatory cytokines such as IL-1 beta and TNF-α could develop encephalopathy by stimulating brain edema, astrocytosis, neutrophil infiltration, and apoptosis. Cerebral endothelial cell activation, reactive oxygen species, and reduced vasodilatory response of the cerebrum are other factors considered in clinical and paraclinical findings in SE. Biopsies in dead patients due to septic shock showed histopathological changes such as cerebral edema, infarcts, microabscesses with intravascular thrombosis, and apoptosis of neurons [51].

Encephalopathy concerning breast cancer represents a consequence of brain metastases. Rising intracranial pressure disturbs cerebral function, which leads to characteristic symptoms of high intracranial pressure. Confusion, memory alterations, headaches, personality disorders, and convulsions are frequently faced in encephalopathy. Moreover, therapies such as chemotherapy or brain radiotherapy could induce the instauration of encephalopathy. The diagnosis and treatment of encephalopathy associated with cancer frequently involve collaboration between oncologists and neurologists. Investigations such as neurologic exams, laboratory tests, and imaging techniques like computed tomography (CT) and magnetic resonance imaging (MRI) are endorsed for cerebral function and identification of the specific cause of symptoms. Treatment is focused on symptom management of the underlying cause, such as tumoral excision or medication to reduce brain inflammation or intracranial pressure. In some cases, radiotherapy may be used to shrink or ablate the tumoral mass to diminish the tumoral pressure.

#### 3.1.4. Management of MODS

The management of MODS in oncological patients requires an optimal multidisciplinary approach in the first place. The specialists need to ensure a comprehensive and integrated manner of coordination regarding MODS; afterward, if the outcome is favorable, oncological therapy can be administered. Therefore, in the following, we will present the management of multiple organ dysfunction syndrome in both oncological and non-oncological patients according to the International Guidelines for Management of Sepsis and Septic Shock on the 2nd of October 2021. Based on the quality of research studies (very low-quality evidence, low-quality evidence, moderate-quality evidence, and high-quality evidence) and the number of pieces of evidence, according to the abovementioned guidelines, actual recommendations are structured in a multilayer fashion (no recommendation, weak recommendation, strong recommendation, best practice statement, weak recommendation against, and strong recommendation against).

Likewise, Asim et al. show in their study that the management of MODS includes antibiotics for sepsis control, microcirculatory and respiratory support for reperfusion, organ-targeted drugs, and the correction of coagulation abnormalities, acid-base imbalances, metabolic issues, and electrolyte imbalances. Furthermore, common predisposing factors with the potential to precipitate MODS necessitate early control or prevention. These factors encompass sepsis/infection, tissue hypoperfusion, microcirculatory failure, and an exacerbated inflammatory response [47].

- Prevention

The acknowledged Latin idea from the 13th century is that facing a problem early is better and more useful than curing it after the damage has been done. For MODS patients, this is no exception. Infection, poor tissue perfusion, and a perpetuating inflammatory status are the most common and essential risk factors for developing MODS. Therefore, in the beginning, therapies should be goal-oriented to prevent or treat early. In addition, as we previously described, the abovementioned risk factors are commonly associated with oncologic patients. Thus, this category of patients has a higher risk than the normal population. Deitch et al. mentioned two decades ago that invasive treatments are important in prolonging survival. However, those treatments are mainly palliative for end-stage organ failure in MODS patients and do not significantly improve the survival rate or the reversibility of the processes leading to organ failure [80].

Different clinical findings and instruments are utilized for screening sepsis, such as SIRS criteria, vital signs, evidence of infection, the qSOFA (quick Sequential Organ Failure) score, and the SOFA (Sequential Organ Failure Assessment) criteria. In addition, Evans et al. describe that machine learning may be a useful tool for the better performance of actual screening mechanisms. The Summary Area Under the Receiver Operating Characteristic Curve (SAUROC) statistical tool was used to measure the predictive accuracy of machine learning versus traditional screening instruments in hospital-acquired sepsis patients. The results emphasized higher specificity and sensitivity in machine learning models than in classical tools [81].

- Conventional Therapies

Antibiotics, antifungals, and antivirals.

In septic shock or elevated chances of sepsis in adult patients, initiation of antimicrobial therapy, preferably in the first hour after admission, should be administered—this is a strong recommendation. In adults with a possibility of sepsis without shock, a quick assignment of the nature of the acute illness (infectious versus non-infectious) should be made—a best practice statement. Empiric antifungal therapy should commence in adults with sepsis or septic shock due to the high risk of fungal infection, while in adults with a reduced risk of fungal infection, no antifungal therapy should be initiated. Neutropenia, immunosuppression, longer ICU length of stay, and severity of illness, such as a high Acute Physiology and Chronic Health Evaluation II (APACHE II) score, are several risk factors that can be identified in oncologic patients and can contribute to Candida sepsis. However, low-quality evidence studies classify this as a weak recommendation. Regarding the antiviral treatment, no recommendations are made.

Hemodynamic management.

Fluid management in adult patients with sepsis or septic shock is recommended to be made with crystalloids as a first-line choice—strong recommendation and moderate quality of evidence—while balanced crystalloids are suggested to be used instead of normal saline resuscitation, with weak recommendation and low quality of evidence. In adults with sepsis or septic shock, albumin is suggested to be joined when extensive volumes of crystalloids are used—weak recommendation but moderate quality of evidence. Starches are not recommended for resuscitation (strong recommendation with high-quality evidence) nor gelatine (weak recommendation, moderate quality of evidence) in adults with sepsis or septic shock.

Norepinephrine, from the class of vasoactive agents, is recommended to be first utilized over other vasopressors in septic shock adults—a strong recommendation. Epinephrine or dopamine can be used as a surrogate of norepinephrine, but all efforts should be made to raise the accessibility of norepinephrine; caution should be exercised in patients at risk for arrhythmias when using epinephrine and dopamine. In inadequate mean arterial pressure (MAP), despite norepinephrine use, vasopressin is suggested to be introduced rather than increasing the dose of norepinephrine (weak recommendation with moderate-quality evidence). In adult patients treated with norepinephrine and vasopressin for septic shock and uncontrolled MAP levels, the guideline suggests the use of epinephrine—a weak recommendation with low-quality evidence. Additionally, terlipressin is advised not to be used in septic shock patients (weak recommendation, low-quality evidence).

Inotropes such as dobutamine, in addition to norepinephrine or epinephrine solely, are recommended to be used in patients with septic shock, cardiac dysfunction, persistent hypoperfusion, and poor control of volume status and arterial blood pressure—a weak recommendation with very low quality of evidence. The present guideline emphasizes against the use of levosimendan in septic shock patients with cardiac dysfunction and refractory hypoperfusion in spite of good volume control status—weak recommendation, low quality of evidence.

Monitoring and intravenous access are suggested to be accomplished if the setting offers that possibility (weak recommendation, very low quality of evidence). Additionally, rather than obtaining central venous access, peripheral vasopressor therapy administered in a vein close to the antecubital fossa is suggested to be initiated to restore MAP (weak recommendation, low quality of evidence).

Regarding fluid balance, there is no sufficient evidence to suggest the use of a defined or a liberal fluid scheme in the first 24 h in patients with sepsis and septic shock who still present signs of reduced blood flow and volume depletion after the first resuscitation. Therefore, fluid resuscitation is reserved for patients who present clear signs of hypoperfusion.

Ventilation.

As to oxygen targets, there is no sufficient evidence to suggest the usage of conservative oxygen targets in sepsis-induced hypoxemic respiratory failure in adults. The guideline suggests high-flow nasal oxygen for adult patients with sepsis-induced hypoxemic respiratory failure. However, this is a weak recommendation with a low quality of evidence.

There is no sufficient evidence to suggest the usage of non-invasive ventilation instead of invasive ventilation in sepsis-induced hypoxemic respiratory failure in adult patients.

With respect to protective ventilation in ARDS, the guideline suggests the usage of a low tidal volume ventilation scheme (6 mL/kg) over a high tidal volume (>10 mL/kg) for adult patients—this recommendation is strong, based on high-quality evidence. Regarding adults with sepsis-induced severe acute respiratory distress syndrome, the guideline suggests the usage of a superior limit goal for plateau pressures of 30 cm H_2_O in comparison to higher plateau pressures—this recommendation is strong, based on moderate quality of evidence. In adult patients with moderate to severe sepsis-induced ARDS, the guideline recommends the usage of a higher PEEP instead of a lower PEEP, although this recommendation is weak with a moderate quality of evidence.

In sepsis-induced respiratory failure (without ARDS) in adult patients, the guideline suggests the usage of a low tidal volume instead of high tidal volume ventilation.

In sepsis-induced moderate–severe acute respiratory distress syndrome in adults, the guideline suggests the usage of the classical recruitment maneuvers—this suggestion is weak but with moderate quality of evidence. In addition, the guideline recommends the usage of prone ventilation for more than 12 h daily for adult patients with sepsis-induced moderate to severe ARDS—this recommendation is strong but with moderate quality of data.

As to the usage of intermittent neuromuscular blocking agents (NMBA), the actual guideline suggests using NMBA boluses instead of NMBA continuous infusion for adult patients with sepsis-induced moderate–severe ARDS—this recommendation is strong but with moderate quality of data.

The guideline suggests the usage of veno-venous (VV) extracorporeal membrane oxygenation (ECMO) for adult patients with sepsis-induced severe ARDS when standard mechanical ventilation fails in expert centers with optimal infrastructure to sustain its work [81].

- Additional Therapies

The guideline suggests the usage of IV corticosteroids for adult patients with septic shock and a continuous demand for vasopressor therapy. The representative corticosteroid used in adult patients with septic shock is IV hydrocortisone with a dosage of 200 mg/day administered as 50 mg intravenously every 6 h or as a constant infusion. The recommendation is that this be begun at a dose of norepinephrine or epinephrine greater than or equal to 0.25 mcg/kg/min for at least 4 h after commencement.

The guideline contraindicates the usage of polymyxin B hemoperfusion in adult patients with sepsis or septic shock. There are no sufficient data to recommend the use of alternative blood purification techniques.

Respecting the usage of a restrictive transfusion strategy in adult patients with sepsis or septic shock, the actual guideline provides a strong recommendation but with moderate-quality data. A selective transfusion approach usually consists of a hemoglobin concentration transfusion trigger of 70 g/L. However, hemoglobin concentration alone should not be the only parameter that guides the red blood cell (RBC) transfusion. The evaluation of patients’ global clinical condition and examination of extenuating circumstances such as acute myocardial ischemia, acute hemorrhage, or severe hypoxemia is mandatory.

As to immunoglobulins, the guideline discourages the usage of intravenous immunoglobulins in adult patients with sepsis or septic shock. Stress ulcer prophylaxis is encouraged in adult patients with sepsis or septic shock who present risk elements for gastrointestinal (GI) bleeding. In addition, pharmacological venous thromboembolism (VTE) prophylaxis is strengthened to be used only if there is no other condition that contraindicates such therapy—this recommendation is strong but based on moderate quality of information. Regarding adult patients with sepsis or septic shock, the guideline indicates therapy with low molecular weight heparin (LMWH) instead of unfractionated heparin (UFH) for VTE prophylaxis. However, the guideline discourages the use of mechanical VTE prophylaxis along with pharmacological prophylaxis in adults with sepsis or septic shock.

Concerning renal replacement therapy (RRT), the guideline suggests either constant or intermittent renal replacement therapy for adult patients with sepsis, septic shock, and AKI who necessitate RRT. In adult patients with no specific indications of RRT who suffer from sepsis, septic shock, or AKI, the guideline discourages renal replacement therapy.

Insulin at a glucose level of 180 mg/dL or higher (10 mmol/L) in adult patients with sepsis or septic shock should be initiated. A standard target blood glucose range is 144–180 mg/dL (8–10 mmol/L) after the beginning of insulin therapy. However, the actual guideline discourages using IV vitamin C in adult patients with sepsis or septic shock, as well as sodium bicarbonate therapy to enhance hemodynamics or to diminish vasopressor requirements for adult patients with hypoperfusion-induced lactic acidemia in sepsis or septic shock. Nonetheless, for adult patients suffering from septic shock, severe metabolic acidemia (pH ≤ 7.2), and acute kidney injury (AKIN score 2 or 3), the guideline suggests treatment with sodium bicarbonate. Early (within 72 h) institution of enteral feeding in adult patients who suffer from sepsis or septic shock who can be fed enterally is advised [81].

In summary, only several actual conventional therapies accomplish the criteria to be classified as high-quality evidence: avoiding starches in resuscitation and protective ventilation in ARDS. While specific therapies lack high-quality evidence in current recommendations, it is essential to note that physicians often observe favorable outcomes with some of these treatments in numerous patients. Therefore, supplementary, high-quality studies need to be designed to improve the actual recommendations.

### 3.2. Quality of Life

During the last decades, the quality of life (QoL) of cancer-diagnosed patients has become a fundamental pillar in the management of the disease because this diagnosis affects patients’ physical, social, and emotional functions. Furthermore, QoL significantly impacts patients’ treatment adherence and their recovery. Engaging in physical and emotional struggles disrupts their day-to-day lives. Aspects such as pain management, psychological comfort, social dynamics, and social independence can profoundly affect patients’ outcomes [82]. Hence, the emphasis on QoL becomes a key objective from the moment of diagnosis [83].

Since the discovery of new therapeutic options in breast cancer [84] (targeted molecular therapy and immunotherapy), a significant increase in survival has been observed in patients with breast cancer. In a study that examined the evolution of survival rates in the last 30 years, an important improvement has been noticed in the five-year survival rate for patients diagnosed with metastatic breast cancer. The study showed an increase in the survival rate by approximately 20 months (from 13 months in 1985 to 33 months in 2016). On the other hand, strategies for care and the improvement of QoL have not developed proportionally to patients’ survival rates [85]. It is a well-known fact for both the patient and the physician that not only the prolongation of survival is important, but the global quality of life as well [86].

The QoL of breast cancer patients can be influenced by multiple factors, on the one hand, by clinical manifestations caused by cancer and, on the other, by the consequences of oncological therapy, such as surgery, chemotherapy, radiotherapy, and endocrine therapy. Depending on the stage of the disease and the type of therapy administered, it has been shown that symptoms like hair loss, pain, lymphedema, nausea, vomiting, fatigue, insomnia, depression, and fear of death have a negative impact on the quality of life [87].

In order to evaluate the quality of life of oncological patients, several questionnaires that addressed many aspects of the patient’s life were created. One of the first QoL questionnaires was developed by EORTC (The European Organization for Research and Treatment of Cancer) in 1993, and since then, it has been constantly updated to the actual version, which is Quality of Life Questionnaire Core 30 (QLQ C30 v3.0). The questionnaire is validated and applied to assess the quality of life for all the patients diagnosed with cancer, both in medical practice and in medical research worldwide. The instrument consists of 30 questions that evaluate several indexes such as global health status, functional scale (physical function, role function, emotional function, cognitive function, and social function), and symptom burden (fatigue, nausea, vomiting, pain, dyspnea, insomnia, appetite loss, constipation, diarrhea, and financial difficulties) [88]. In the context of breast cancer, the evaluation involves applying the QLQ-C30 questionnaire with an additional module, Quality of Life Questionnaire—Breast Cancer (QLQ-BR45). The QLQ-BR45 represents a specific survey for breast cancer, which evaluates, in addition, the functional item (body image, sexual functioning, sexual enjoyment, future perspective, breast satisfaction), symptom items (systemic therapy side effects, arm symptoms, breast symptoms, upset by hair loss), and target therapy scale (hormonal therapy symptoms and endocrine sexual symptoms) [89]. Another questionnaire with a specific appeal in the evaluation of the quality of life for patients diagnosed with breast cancer is Functional Assessment of Cancer Therapy—Breast (FACT-B), which establishes physical, functional, emotional, social, and additional well-being. The easy manner in which to apply the questionnaire, its reliability, and its sensitivity make FACT-B useful in both medical practice and research [90]. The Short Form 36 Health Survey (SF-36) is another instrument utilized to assess the quality of life by evaluating eight interest fields: limitations regarding physical and social activities, the role of the patient due to physical and emotional impairment, pain, mind health, vitality, and perceptions about the general health status [91]. Mertz et al. suggested that QoL should become a primary co-objective in clinical studies to establish the impact of new therapies on the quality of life of oncological patients. Moreover, the quality of life of patients admitted to clinical trials should be evaluated continuously through the questionnaires mentioned above [86,92].

For ICU-hospitalized patients, a series of prognosis scores are created to evaluate the severity of MODS. A well-known score is SOFA—utilizing it determines the grade of respiratory, cardiovascular, hepatic, renal, neurological, and coagulation dysfunction. In the case of breast cancer patients admitted to the ICU, it was discovered that a high SOFA score stands for mortality risk factor during admission, together with high levels of alanine aminotransferase (ALT) and cardiovascular complications [93]. Another score that appraises the prognosis of breast cancer patients hospitalized in the ICU is the APACHE II score. This score is settled in the first 24 h after the ICU admission by the evaluation of mean arterial pressure, heart rate, temperature, respiratory rate, oxygenation, arterial pH, serum sodium, potassium, creatinine, mean corpuscular volume, leucocytes, age, Glasgow Coma Score, and chronic organ disease. A study conducted by Headley et al. presented a correlation between the APACHE II score and mortality in breast cancer patients admitted to the ICU. In-hospital mortality was associated with both the number of metastatic sites and major organ system failure. Therefore, for patients experiencing more than two metastatic sites and significant organ system failure or respiratory failure, the survival rate was markedly diminished [45].

In 2021, Moscetti et al. approached several aspects regarding the quality of life of patients who received hormonal treatment in comparison with those who underwent standard therapy (two groups of patients with a percentage of similarity of about 40%). The study emphasized that patients who received chemotherapy registered a higher level of anxiety/depression in comparison with patients who received endocrine therapy, leading to the conclusion that the latter had a higher quality of life than those who received standard chemotherapy [94]. In addition, a study conducted by Hamer et al. in 2016 with the aim of determining the quality of life and the severity of the symptoms in breast cancer patients underlines the fact that metastatic breast cancer patients who underwent 25-fraction radiation (5000 cGy/25) registered a lower quality of life and a greater severity of symptoms than those who received 16-fraction radiation (4256 cGy/16). In the same study, supplementary tests were conducted to determine the impact of radiotherapy on quality of life. The first analysis compared patients who underwent radiation therapy alone with patients who received no therapy at all and showed that patients who received radiotherapy registered a greater severity of symptoms, like shortness of breath. Also, it is essential to acknowledge that there were no differences registered between the groups that only received hormone therapy and the groups that received both hormone therapy and radiotherapy [95].

An important aspect of oncological patients’ care is represented by the admission of these patients to intensive care units, a controversial decision that is not strongly guided by the actual therapy protocols. Biskup et al. emphasize that this decision is frequently influenced by a range of significant factors, including life expectancy and quality of life in the absence of acute illness, the estimated duration of ICU hospitalization, the post-ICU rehabilitation period, the implications of the critical disease on ongoing oncological therapy, and its subsequent consequences. Additionally, specific interactions between conditions induced by cancer and cancer therapy, combined with ICU management, play a key role. Furthermore, the patient’s preferences and wishes contribute substantially to this decision-making process [96]. However, there is a thin balance between the benefits of specific ICU care and their associated risks. Therefore, future studies should focus on the identification of prognosis markers and evidence-based criteria regarding the selection of patients for admission to the ICU, improving both the results and the quality of care for this vulnerable category of patients.

Despite the existence of a wide range of treatments and optimal therapeutic regimens based on the associated comorbidities of each patient, the quality of life of metastatic cancer patients remains unchanged. Exceptionally, O’Shaughnessy mentions in his work the fact that patients who underwent Trastuzumab therapy may have a slightly better quality of life, provided their cardiac function is regularly inspected with accuracy [97].

### 3.3. Analysis of AI-Based Clustering

**Figure 4:**
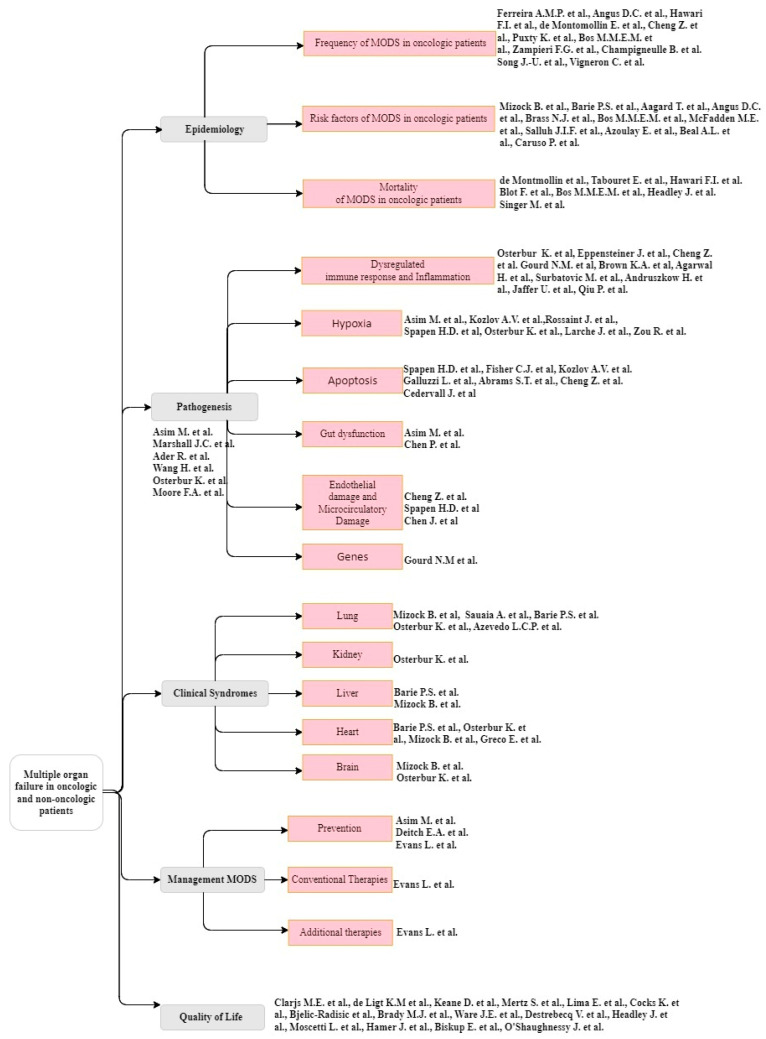
Manual taxonomy was used in our manuscript preparation. Each article is identified by the main author’s name (References are arranged in a descending order from top to bottom. Frequency of MODS in oncologic patients: [24,25,26,29,30,31,32,33,34,68]; Risk factors of MODS in oncologic patients: [1,25,30,35,36,37,38,39,40,41,42]; Mortality of MODS in oncologic patients: [26,27,30,43,44,45]; Dysregulated immune response and Inflammation: [2,51,54,55,56,57,58,59,60,69]; Hypoxia: [47,51,61,62,63,64,65]; Apoptosis: [28,61,63,66,68,69,70]; Pathogenesis: [46,47,48,49,50,51]; Gut dysfunction: [46,70]; Endothelial damage and Microcirculatory damage: [62,68,73]; Genes: [2]; Lung: [35,36,50,75,76]; Kidney: [50]; Liver: [35,36]; Heart: [35,36,50,78]; Brain: [35,50]; Prevention: [46,79,80]; Conventional Therapies: [80]; Additional therapies: [80]; Quality of Life: [44,81,82,84,85,86,87,88,89,90,92,93,94,95]).

We added the name of the first author of each scientific article used in our work to each subchapter of the taxonomy, which served as a pattern to construct our narrative review, according to Figure 4. Thus, we had a visual record of the articles that were grouped and ordered according to the review structure.

**Figure 5:**
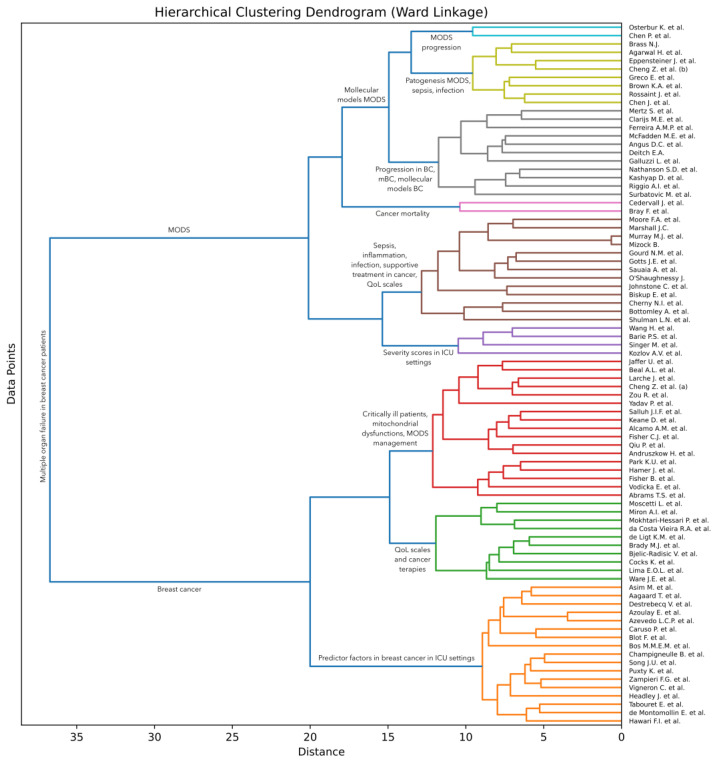
AI system dendrogram. *X*-axis (horizontal): the *X*-axis represents the distance or dissimilarity between different clusters of articles. As we move from 35 to 0 along the *X*-axis, the similarity between clusters increases, which means that the AI system searches for deep similarities between articles. We used a distance threshold of 13 to color the clusters. *Y*-axis (vertical): the *Y*-axis represents the data points (articles) in our dendrogram. Each data point is positioned vertically based on its cluster or subgroup. Clusters: articles that are close to each other on the dendrogram are more similar to each other, while articles that are farther apart are less similar. The clusters formed in the dendrogram indicate groups of articles with similar characteristics or attributes (References are arranged in a descending order from top to bottom: [1,2,5,6,9,10,11,12,23,24,25,26,27,28,29,30,31,32,33,34,35,36,37,38,39,40,41,42,43,44,45,46,47,49,50,51,53,54,55,56,58,60,61,63,64,65,66,67,68,69,70,73,75,76,78,79,81,82,84,85,86,87,88,89,90,92,93,95,96,98]).

Later, we turned to a clustering analysis system based on artificial intelligence to group the retrieved articles into distinct categories so that the items in each group are more similar to each other than to the items in the other groups. This analysis begins with individual articles and gradually merges them into larger groups based on the similarities observed between the articles. The algorithm is performed until all the articles are joined into one large group. This is illustrated in the dendrogram shown in Figure 5. It is important to mention that this analysis was performed based on the titles of the articles and their abstracts, not on the full version of the articles. This is a limitation of the BERT model [20], which accepts at most 512 tokens (words) per article as input. We underline that this limitation does not have a major impact on the resulting dendrogram since the abstracts are expected to accurately represent the content of the surveyed articles. This was also observed by Soviany et al. [18], who clustered the articles in their survey based on the abstracts.

The dendrogram shown in Figure 5 is the result of an unsupervised algorithm that clusters articles based on the similarity among BERT embeddings. Since the BERT model is a pre-trained language model, it can accurately represent the global similarity of any two text samples. Still, the clustering algorithm is not particularly tuned to the domain of interest, which might lead to generating clusters that are not representative of the studied domain. We note that this limitation is not particular to our clustering algorithm, as it generally applies to all unsupervised methods. To overcome this limitation, the validity of the resulting dendrogram must be manually verified by experts. To this end, we conducted a manual analysis of the dendrogram presented in Figure 5, corroborating the findings with the handmade dendrogram shown in Figure 4.

Analyzing the resulting dendrogram in Figure 5, we can conclude that the AI system has grouped our items quite non-homogeneously in certain categories compared to our handmade dendrogram (see Figure 4). For example, articles from the clinical syndrome subchapter were grouped into other subchapters (Osterbur K. et al. [51] was manually clustered in our taxonomy in the kidney category, while the AI system grouped it in MODS progression in Figure 5). This could mean either that AI has observed certain similarities that the human mind, due to reading many articles, did not notice, or that the abstracts of articles did not fully emphasize the most relevant aspects considered in the construction of the manual taxonomy. As far as we observed during manual selection, the term “progression” was used in both MODS progression and breast cancer progression. Thus, the AI-based system created a cluster for MODS progression (a light blue cluster with articles from Osterbur K. et al. [51] to Chen P. et al. [71].) and a cluster with BC progression (a gray cluster with articles from Mertz S. et al. [86]. to Surbatovic M. et al. [57]). Then, the AI model grouped them both into a new big cluster.

In the light green cluster (from Brass N. J. [39] to Chen J. et al. [74]), the AI model grouped articles containing information about the pathogenesis of MODS, sepsis, infection, and molecular models of MODS. In the gray cluster (from Mertz S. et al. [86] to Surbatovic M. et al. [57]), it grouped progression in BC, mBC, and molecular models BC. In the pink cluster (Cedervall J. et al. [70]), the AI model united information about cancer mortality. The brown cluster contains information about sepsis, inflammation, infection, supportive treatment for cancer, and quality of life scales. Notably, one thing that went unnoticed by the human eye and the AI system was the very similarity between the abstracts of articles published by Murray M.J. et al. [99] and Mizock B. [35], illustrated in this cluster. Severity scores in ICU settings were joined in the purple cluster (from Wang H. et al. [50] to Kozlov A.V. et al. [61]). Critically ill patients, mitochondrial dysfunctions, and MODS management were grouped into the red cluster (from Jaffer U. et al. [59] to Abrams T.S. et al. [68]). The green cluster (from Moscetti L. et al. [94] to Ware J.E. et al. [91]) grouped articles containing information about QoL scales and cancer therapies, and the orange cluster (from Asim M. et al. [47] to Hawari F.I. et al. [27]) grouped information about predictor factors in breast cancer patients in ICU settings. The AI model then created two large clusters with MODS and another with breast cancer, uniting them into a single large cluster—multiple organ failure in breast cancer patients.

In summary, our analysis shows that by using AI models, we can find aspects overlooked by the human eye, which could represent a direction of research in identifying subclinical organ failure syndrome.

## 4. Future Directions

Further studies are required to offer deep knowledge and validate the concept of the subclinical syndrome of multiple organ failure in oncologic patients as well as in non-oncologic patients. Moreover, it is important to find the answer to the question “Why do patients degrade?” and how their biological status evolves from subclinical multiple organ failure syndrome to acute (clinical) multiple organ failure that resembles the effect of dominoes falling. Restricting the progression to multiple organ failure, enhancing the quality of life, optimizing non-oncological treatment, and extending overall survival represent supplementary yet indispensable objectives. We anticipate that forthcoming studies, possible with the aid of data-driven artificial intelligence, will uncover the interdependencies between the breast cancer tumor stage and the compensatory changes undergone inside the body to maintain homeostasis. Understanding these dynamics is crucial for preventing the chain decompensation of subsequent affected organs. We note that AI has already been employed in cancer detection [98], providing assistance to physicians around the world. Once sufficient data about the subclinical syndrome of organ insufficiency are gathered, AI-based techniques can be employed in future work to learn patterns from these data, which could lead to the discovery of automatic tools for diagnosing this syndrome. We consider that deep learning techniques are more likely to prevail in this future endeavor since such methods have recently revolutionized the field of AI.

On a distinct note, we underline that the proposed clustering algorithm is generic. Hence, researchers can employ it without any change in their future work to obtain clusters of articles, regardless of the surveyed domain. The AI-based algorithm can reveal unexpected clusters of articles, which could be overlooked when manually constructing a taxonomy of articles.

## 5. Conclusions

The purpose of the present research was to propose a novel perspective on subclinical organ failure syndrome, multiple organ failure, and breast cancer. Both the precursor lesions of breast cancer and the subclinical syndrome of multiple organ failure are early stages of some diseases in which changes are expressed before the symptoms become clinical or identifiable by the usual diagnostic methods. The modifications in pathophysiology, which can start from the genetic level and up to the clinical manifestation, make MODS evolve from a subclinical syndrome of organ failure through the domino effect up to an acute, clinical, multiple organ failure, resulting in severe disease or even death. Both conditions usually evolve through stages, going through a progressive deterioration process. Thus, as we diagnose and treat cancer progressively, it would be ideal to be able to diagnose and treat MODS. The major limitation of this study is that the actual literature fails to give sufficient consideration to the existence of subclinical multiple organ failure syndrome and MODS in breast cancer patients. Notwithstanding the relatively limited data, this work offers valuable insights into specific genes, molecular markers, and biochemical parameters associated with this phenomenon. Accordingly, we propose initiating clinical studies on patients to observe the real-world staging of MODS linked to breast cancer. This pragmatic approach will enhance the external validity and applicability of findings, contributing to a more comprehensive understanding of the complex connection between cancer and multiple organ dysfunction in the clinical settings faced by patients and healthcare providers.

The review also investigates the data on the epidemiology, pathogenesis, clinical syndromes, and management of oncologic patients with MODS. Pathogenesis pathways have been extensively studied recently, seeking a deeper understanding of cells and molecules that could benefit from novel therapies. The results highlight a lack of significant improvements in MODS management, emphasizing the ongoing need for research to provide adequate treatment, halt the chain degradation process in organ systems, enhance both oncological and non-oncological treatment, and elevate the quality of life. Despite efforts, the quality of life among metastatic cancer patients remains unchanged. A critical aspect of caring for oncological patients involves decisions regarding admission to intensive care units. This matter lacks clear guidance from existing therapy protocols, rendering it a contentious issue. Future research should focus on identifying prognostic markers and establishing evidence-based criteria for patient selection in ICU admission. This approach aims to improve both outcomes and the quality of care for this vulnerable patient population.

While the existence of the subclinical organ failure syndrome is acknowledged, current diagnostic methodologies prove insufficient for its accurate identification. Consequently, we conjecture that the increasing significance of AI will emerge as a pivotal tool, empowering physicians to diagnose this syndrome effectively.

## References

[1] McFadden M.E., Sartorius S.E. (1992). Multiple Systems Organ Failure in the Patient with Cancer. Part I: Pathophysiologic Perspectives. Oncol. Nurs. Forum.

[2] Gourd N.M., Nikitas N. (2020). Multiple Organ Dysfunction Syndrome. J. Intensive Care Med..

[3] Fisher B., Jeong J.H., Dignam J., Anderson S., Mamounas E., Wickerham D.L., Wolmark N. (2001). Findings from Recent National Surgical Adjuvant Breast and Bowel Project Adjuvant Studies in Stage I Breast Cancer. J. Natl. Cancer Inst. Monogr..

[4] Coniac S., Costache Outas M.C., Pirvu E.-E., Patru R.-I., Gainariu E., Aldea C., Iorga P.G., Ambroci M., Liscu H.-D., Miron A.-I. (2023). Challenges and Limitations of Endocrine Toxicity Evaluation in Non-Small Cell Lung Cancer Patients Treated with Immunotherapy—Retrospective Study from a Tertiary-Level Hospital in Romania. Diagnostics.

[5] Miron A.-I., Anghel A.-V., Barnonschi A.-A., Mitre R., Liscu H.-D., Găinariu E., Pătru R., Coniac S. (2023). Real-World Outcomes of CDK4/6 Inhibitors Treatment in Metastatic Breast Cancer in Romania. Diagnostics.

[6] Nathanson S.D., Detmar M., Padera T.P., Yates L.R., Welch D.R., Beadnell T.C., Scheid A.D., Wrenn E.D., Cheung K. (2022). Mechanisms of Breast Cancer Metastasis. Clin. Exp. Metastasis.

[7] Park K.U., Chen Y., Chitale D., Choi S., Ali H., Nathanson S.D., Bensenhaver J., Proctor E., Petersen L., Loutfi R. (2018). Utilization of the 21-Gene Recurrence Score in a Diverse Breast Cancer Patient Population: Development of a Clinicopathologic Model to Predict High-Risk Scores and Response to Neoadjuvant Chemotherapy. Ann. Surg. Oncol..

[8] Cardoso F., Paluch-Shimon S., Senkus E., Curigliano G., Aapro M.S., André F., Barrios C.H., Bergh J., Bhattacharyya G.S., Biganzoli L. (2020). 5th ESO-ESMO International Consensus Guidelines for Advanced Breast Cancer (ABC 5). Ann. Oncol..

[9] Vodicka E., Kim K., Devine E.B., Gnanasakthy A., Scoggins J.F., Patrick D.L. (2015). Inclusion of Patient-Reported Outcome Measures in Registered Clinical Trials: Evidence from ClinicalTrials.Gov (2007–2013). Contemp. Clin. Trials.

[10] Bottomley A., Reijneveld J.C., Koller M., Flechtner H., Tomaszewski K.A., Greimel E. (2019). 5th EORTC Quality of Life in Cancer Clinical Trials Conference Faculty Current State of Quality of Life and Patient-Reported Outcomes Research. Eur. J. Cancer.

[11] Cherny N.I., Sullivan R., Dafni U., Kerst J.M., Sobrero A., Zielinski C., de Vries E.G.E., Piccart M.J. (2015). A Standardised, Generic, Validated Approach to Stratify the Magnitude of Clinical Benefit That Can Be Anticipated from Anti-Cancer Therapies: The European Society for Medical Oncology Magnitude of Clinical Benefit Scale (ESMO-MCBS). Ann. Oncol..

[12] Mokhtari-Hessari P., Montazeri A. (2020). Health-Related Quality of Life in Breast Cancer Patients: Review of Reviews from 2008 to 2018. Health Qual Life Outcomes.

[13] Aggarwal C., Reddy C. (2014). Data Clustering: Algorithms and Applications.

[14] Ristea N.-C., Miron A.-I., Savencu O., Georgescu M.-I., Verga N., Khan F.S., Ionescu R.T. (2023). CyTran: A Cycle-Consistent Transformer with Multi-Level Consistency for Non-Contrast to Contrast CT Translation. Neurocomputing.

[15] Georgescu M.-I., Ionescu R.T., Miron A.-I., Savencu O., Ristea N.-C., Verga N., Khan F.S. Multimodal Multi-Head Convolutional Attention with Various Kernel Sizes for Medical Image Super-Resolution. Proceedings of the 2023 IEEE/CVF Winter Conference on Applications of Computer Vision (WACV).

[16] Georgescu M.-I., Ionescu R.T., Miron A.I. (2023). Diversity-Promoting Ensemble for Medical Image Segmentation. Proceedings of the Proceedings of the 38th ACM/SIGAPP Symposium on Applied Computing.

[17] Ionescu R.T., Popescu M., Ionescu R.T., Popescu M. (2016). Learning Based on Similarity. Knowledge Transfer between Computer Vision and Text Mining: Similarity-based Learning Approaches.

[18] Soviany P., Ionescu R.T., Rota P., Sebe N. (2022). Curriculum Learning: A Survey. Int. J. Comput. Vis..

[19] Ward J.H. (1963). Hierarchical Grouping to Optimize an Objective Function. J. Am. Stat. Assoc..

[20] Devlin J., Chang M.-W., Lee K., Toutanova K., Burstein J., Doran C., Solorio T. (2019). BERT: Pre-Training of Deep Bidirectional Transformers for Language Understanding. Proceedings of the 2019 Conference of the North American Chapter of the Association for Computational Linguistics: Human Language Technologies, Volume 1 (Long and Short Papers).

[21] BERT. https://huggingface.co/docs/transformers/model_doc/bert.

[22] Scarlat F., Scarisoreanu A., Verga N. (2013). Absorbed dose distributions using the isodensitometric method for exposures with filter employed for mammographies. Rom. Rep. Phys..

[23] Alcamo A.M., Pang D., Bashir D.A., Carcillo J.A., Nguyen T.C., Aneja R.K. (2019). Role of Damage-Associated Molecular Patterns and Uncontrolled Inflammation in Pediatric Sepsis-Induced Multiple Organ Dysfunction Syndrome. J. Pediatr. Intensive Care.

[24] Ferreira A.M.P., Sakr Y. (2011). Organ Dysfunction: General Approach, Epidemiology, and Organ Failure Scores. Semin. Respir. Crit. Care Med..

[25] Angus D.C., Linde-Zwirble W.T., Lidicker J., Clermont G., Carcillo J., Pinsky M.R. (2001). Epidemiology of Severe Sepsis in the United States: Analysis of Incidence, Outcome, and Associated Costs of Care. Crit. Care Med..

[26] Hawari F.I., Nazer L.H., Addassi A., Rimawi D., Jamal K. (2016). Predictors of ICU Admission in Patients with Cancer and the Related Characteristics and Outcomes: A 5-Year Registry-Based Study. Crit. Care Med..

[27] de Montmollin E., Tandjaoui-Lambiotte Y., Legrand M., Lambert J., Mokart D., Kouatchet A., Lemiale V., Pène F., Bruneel F., Vincent F. (2013). Outcomes in critically ill cancer patients with septic shock of pulmonary origin. Shock.

[28] Cheng Z., Abrams S.T., Toh J., Wang S.S., Wang Z., Yu Q., Yu W., Toh C.-H., Wang G. (2020). The Critical Roles and Mechanisms of Immune Cell Death in Sepsis. Front. Immunol..

[29] Puxty K., McLoone P., Quasim T., Sloan B., Kinsella J., Morrison D.S. (2015). Risk of Critical Illness Among Patients With Solid Cancers: A Population-Based Observational Study. JAMA Oncol..

[30] Bos M.M.E.M., Verburg I.W.M., Dumaij I., Stouthard J., Nortier J.W.R., Richel D., van der Zwan E.P.A., de Keizer N.F., de Jonge E. (2015). Intensive Care Admission of Cancer Patients: A Comparative Analysis. Cancer Med..

[31] Zampieri F.G., Romano T.G., Salluh J.I.F., Taniguchi L.U., Mendes P.V., Nassar A.P., Costa R., Viana W.N., Maia M.O., Lima M.F.A. (2021). Trends in Clinical Profiles, Organ Support Use and Outcomes of Patients with Cancer Requiring Unplanned ICU Admission: A Multicenter Cohort Study. Intensive Care Med..

[32] Champigneulle B., Merceron S., Lemiale V., Geri G., Mokart D., Bruneel F., Vincent F., Perez P., Mayaux J., Cariou A. (2015). What is the outcome of cancer patients admitted to the ICU after cardiac arrest? Results from a multicenter study. Resuscitation.

[33] Song J.-U., Suh G.Y., Chung M.P., Kim H., Kwon O.J., Jung C.W., Kang W.K., Park K., Jeon K. (2011). Risk Factors to Predict Outcome in Critically Ill Cancer Patients Receiving Chemotherapy in the Intensive Care Unit. Support. Care Cancer.

[34] Vigneron C., Charpentier J., Coussy F., Alexandre J., Pène F., Jamme M. (2023). When Breast Cancer Comes to the ICU: Outcomes and Prognostic Factors. Acta Oncol..

[35] Mizock B. (2009). The Multiple Organ Dysfunction Syndrome. Dis.-a-Mon. DM.

[36] Barie P.S., Hydo L.J. (2000). Epidemiology of Multiple Organ Dysfunction Syndrome in Critical Surgical Illness. Surg. Infect..

[37] Aagaard T., Reekie J., Jørgensen M., Roen A., Daugaard G., Specht L., Sengeløv H., Mocroft A., Lundgren J., Helleberg M. (2020). Mortality and Admission to Intensive Care Units after Febrile Neutropenia in Patients with Cancer. Cancer Med..

[38] Brass N.J. (1994). Predisposition to Multiple Organ Dysfunction. Crit. Care Nurs. Q..

[39] Salluh J.I.F., Soares M., De Meis E. (2009). Antiphospholipid Antibodies and Multiple Organ Failure in Critically Ill Cancer Patients. Clinics.

[40] Azoulay E., Lemiale V., Mokart D., Pène F., Kouatchet A., Perez P., Vincent F., Mayaux J., Benoit D., Bruneel F. (2014). Acute Respiratory Distress Syndrome in Patients with Malignancies. Intensive Care Med..

[41] Beal A.L. (1994). Multiple Organ Failure Syndrome in the 1990s: Systemic Inflammatory Response and Organ Dysfunction. JAMA.

[42] Caruso P., Ferreira A.C., Laurienzo C.E., Titton L.N., Terabe D.S.M., Carnieli D.S., Deheinzelin D. (2010). Short- and Long-Term Survival of Patients with Metastatic Solid Cancer Admitted to the Intensive Care Unit: Prognostic Factors. Eur. J. Cancer Care.

[43] Tabouret E., Boucard C., Devillier R., Barrie M., Boussen S., Autran D., Chinot O., Bruder N. (2016). Neuro-Oncological Patients Admitted in Intensive-Care Unit: Predictive Factors and Functional Outcome. J. Neurooncol.

[44] Blot F., Guiguet M., Nitenberg G., Leclercq B., Gachot B., Escudier B. (1997). Prognostic Factors for Neutropenic Patients in an Intensive Care Unit: Respective Roles of Underlying Malignancies and Acute Organ Failures. Eur. J. Cancer.

[45] Headley J., Theriault R., Smith T.L. (1992). Independent Validation of APACHE II Severity of Illness Score for Predicting Mortality in Patients with Breast Cancer Admitted to the Intensive Care Unit. Cancer.

[46] Singer M., Deutschman C.S., Seymour C.W., Shankar-Hari M., Annane D., Bauer M., Bellomo R., Bernard G.R., Chiche J.-D., Coopersmith C.M. (2016). The Third International Consensus Definitions for Sepsis and Septic Shock (Sepsis-3). JAMA.

[47] Asim M., Amin F., El-Menyar A. (2020). Multiple Organ Dysfunction Syndrome: Contemporary Insights on the Clinicopathological Spectrum. Qatar Med. J..

[48] Marshall J.C. (2001). Inflammation, Coagulopathy, and the Pathogenesis of Multiple Organ Dysfunction Syndrome. Crit. Care Med..

[49] Ader R., Cohen N., Felten D. (1995). Psychoneuroimmunology: Interactions between the Nervous System and the Immune System. Lancet.

[50] Wang H., Ma S. (2008). The Cytokine Storm and Factors Determining the Sequence and Severity of Organ Dysfunction in Multiple Organ Dysfunction Syndrome. Am. J. Emerg. Med..

[51] Osterbur K., Mann F.A., Kuroki K., DeClue A. (2014). Multiple Organ Dysfunction Syndrome in Humans and Animals. J. Vet. Intern. Med..

[52] Moore F.A., Moore E.E. (1995). Evolving Concepts in the Pathogenesis of Postinjury Multiple Organ Failure. Surg. Clin. N. Am..

[53] Groza A., Iconaru S.L., Jiga G., Chapon P., Gaiaschi S., Verga N., Beuran M., Prodan A.M., Matei M., Marinescu S.A. (2019). The Effect of the Ionizing Radiation on Hydroxyapatite–Polydimethylsiloxane Layers. Polym. Eng. Sci..

[54] Eppensteiner J., Kwun J., Scheuermann U., Barbas A., Limkakeng A.T., Kuchibhatla M., Elster E.A., Kirk A.D., Lee J. (2019). Damage- and Pathogen-Associated Molecular Patterns Play Differential Roles in Late Mortality after Critical Illness. JCI Insight.

[55] Brown K.A., Brain S.D., Pearson J.D., Edgeworth J.D., Lewis S.M., Treacher D.F. (2006). Neutrophils in Development of Multiple Organ Failure in Sepsis. Lancet.

[56] Agarwal H., Reddy S., Sharma K. (2014). Aberrant Cellular Signaling in Multiple Organ Failure: Mechanism, Consequences and Therapeutic Applications. J. Drug Deliv. Ther..

[57] Surbatovic M., Veljovic M., Jevdjic J., Popovic N., Djordjevic D., Radakovic S. (2013). Immunoinflammatory Response in Critically Ill Patients: Severe Sepsis and/or Trauma. Mediat. Inflamm..

[58] Andruszkow H., Fischer J., Sasse M., Brunnemer U., Andruszkow J.H.K., Gänsslen A., Hildebrand F., Frink M. (2014). Interleukin-6 as Inflammatory Marker Referring to Multiple Organ Dysfunction Syndrome in Severely Injured Children. Scand. J. Trauma Resusc. Emerg. Med..

[59] Jaffer U., Wade R.G., Gourlay T. (2010). Cytokines in the Systemic Inflammatory Response Syndrome: A Review. HSR Proc. Intensive Care Cardiovasc. Anesth..

[60] Qiu P., Cui X., Sun J., Welsh J., Natanson C., Eichacker P.Q. (2013). Antitumor Necrosis Factor Therapy Is Associated with Improved Survival in Clinical Sepsis Trials: A Meta-Analysis. Crit. Care Med..

[61] Kozlov A.V., Grillari J. (2022). Pathogenesis of Multiple Organ Failure: The Impact of Systemic Damage to Plasma Membranes. Front. Med..

[62] Rossaint J., Zarbock A. (2015). Pathogenesis of Multiple Organ Failure in Sepsis. Crit. Rev. Immunol..

[63] Spapen H.D., Jacobs R., Honoré P.M. (2017). Sepsis-Induced Multi-Organ Dysfunction Syndrome—A Mechanistic Approach. J. Emerg. Crit. Care Med..

[64] Larche J., Lancel S., Hassoun S.M., Favory R., Decoster B., Marchetti P., Chopin C., Neviere R. (2006). Inhibition of Mitochondrial Permeability Transition Prevents Sepsis-Induced Myocardial Dysfunction and Mortality. J. Am. Coll. Cardiol..

[65] Zou R., Tao J., Qiu J., Lu H., Wu J., Zhu H., Li R., Mui D., Toan S., Chang X. (2022). DNA-PKcs promotes sepsis-induced multiple organ failure by triggering mitochondrial dysfunction. J. Adv. Res..

[66] Fisher C.J., Agosti J.M., Opal S.M., Lowry S.F., Balk R.A., Sadoff J.C., Abraham E., Schein R.M., Benjamin E. (1996). Treatment of Septic Shock with the Tumor Necrosis Factor Receptor:Fc Fusion Protein. The Soluble TNF Receptor Sepsis Study Group. N. Engl. J. Med..

[67] Galluzzi L., Vitale I., Aaronson S.A., Abrams J.M., Adam D., Agostinis P., Alnemri E.S., Altucci L., Amelio I., Andrews D.W. (2018). Molecular Mechanisms of Cell Death: Recommendations of the Nomenclature Committee on Cell Death 2018. Cell Death Differ.

[68] Abrams S.T., Morton B., Alhamdi Y., Alsabani M., Lane S., Welters I.D., Wang G., Toh C.H. (2019). A Novel Assay for Neutrophil Extracellular Trap Formation Independently Predicts Disseminated Intravascular Coagulation and Mortality in Critically Ill Patients. Am. J. Respir. Crit. Care Med..

[69] Cheng Z., Abrams S.T., Alhamdi Y., Toh J., Yu W., Wang G., Toh C.-H. (2019). Circulating Histones Are Major Mediators of Multiple Organ Dysfunction Syndrome in Acute Critical Illnesses. Crit. Care Med..

[70] Cedervall J., Zhang Y., Olsson A.-K. (2016). Tumor-Induced NETosis as a Risk Factor for Metastasis and Organ Failure. Cancer Res..

[71] Chen P. (2020). Gut Microbiota and Pathogenesis of Organ Injury.

[72] Petca A., Miron B.C., Pacu I., Dumitrașcu M.C., Mehedințu C., Șandru F., Petca R.-C., Rotar I.C. (2022). HELLP Syndrome-Holistic Insight into Pathophysiology. Medicina.

[73] Sandru F., Petca R.-C., Costescu M., Dumitrașcu M.C., Popa A., Petca A., Miulescu R.-G. (2021). Cutaneous Mastocytosis in Childhood—Update from the Literature. J. Clin. Med..

[74] Chen J., Wei H. (2021). Immune Intervention in Sepsis. Front. Pharmacol..

[75] Teodorescu C.O.D., Șandru F., Charkaoui A., Teodorescu A., Popa A.R., Miron A.-I. (2021). The Dynamic Changes in the Pattern of Liver Function Tests in Pregnant Obese Women. Exp. Ther. Med..

[76] Sauaia A., Moore F.A., Moore E.E. (2017). Postinjury Inflammation and Organ Dysfunction. Crit. Care Clin..

[77] Azevedo L.C.P., Caruso P., Silva U.V.A., Torelly A.P., Silva E., Rezende E., Netto J.J., Piras C., Lobo S.M.A., Knibel M.F. (2014). Outcomes for Patients with Cancer Admitted to the ICU Requiring Ventilatory Support: Results from a Prospective Multicenter Study. Chest.

[78] Sandru F., Petca A., Dumitrascu M.C., Petca R.C., Carsote M. (2021). Peutz-Jeghers syndrome: Skin manifestations and endocrine anomalies (Review). Exp. Ther. Med..

[79] Greco E., Lupia E., Bosco O., Vizio B., Montrucchio G. (2017). Platelets and Multi-Organ Failure in Sepsis. Int. J. Mol. Sci..

[80] Deitch E.A. (1992). Multiple Organ Failure. Pathophysiology and Potential Future Therapy. Ann. Surg..

[81] Evans L., Rhodes A., Alhazzani W., Antonelli M., Coopersmith C.M., French C., Machado F.R., Mcintyre L., Ostermann M., Prescott H.C. (2021). Surviving Sepsis Campaign: International Guidelines for Management of Sepsis and Septic Shock 2021. Intensive Care Med..

[82] Clarijs M.E., Thurell J., Kühn F., Uyl-de Groot C.A., Hedayati E., Karsten M.M., Jager A., Koppert L.B. (2021). Measuring Quality of Life Using Patient-Reported Outcomes in Real-World Metastatic Breast Cancer Patients: The Need for a Standardized Approach. Cancers.

[83] de Ligt K.M., de Rooij B.H., Hedayati E., Karsten M.M., Smaardijk V.R., Velting M., Saunders C., Travado L., Cardoso F., Lopez E. (2023). International Development of a Patient-Centered Core Outcome Set for Assessing Health-Related Quality of Life in Metastatic Breast Cancer Patients. Breast Cancer Res. Treat..

[84] Liscu H.-D., Liscu B.-R., Mitre R., Anghel I.-V., Antone-Iordache I.-L., Balan A., Coniac S., Miron A.-I., Halcu G. (2023). The Conditioning of Adjuvant Chemotherapy for Stage II and III Rectal Cancer Determined by Postoperative Pathological Characteristics in Romania. Medicina.

[85] Keane D., Phillips G., Mitchell N., Connolly R.M., Hegarty J. (2023). Improving Quality of Life and Symptom Experience in Patients with Metastatic Breast Cancer: A Systematic Review of Supportive Care Interventions. Psychooncology.

[86] Mertz S., Benjamin C., Girvalaki C., Cardone A., Gono P., May S.G., Comerford E., Than K.-S., Birch K., Roach M. (2022). Progression-Free Survival and Quality of Life in Metastatic Breast Cancer: The Patient Perspective. Breast.

[87] Lima E.d.O.L., Silva M.M. (2020). da Quality of Life of Women with Locally Advanced or Metastatic Breast Cancer. Rev. Gauch. Enferm..

[88] Cocks K., Wells J.R., Johnson C., Schmidt H., Koller M., Oerlemans S., Velikova G., Pinto M., Tomaszewski K.A., Aaronson N.K. (2023). Content Validity of the EORTC Quality of Life Questionnaire QLQ-C30 for Use in Cancer. Eur. J. Cancer.

[89] Bjelic-Radisic V., Cardoso F., Cameron D., Brain E., Kuljanic K., da Costa R.A., Conroy T., Inwald E.C., Serpentini S., Pinto M. (2020). An International Update of the EORTC Questionnaire for Assessing Quality of Life in Breast Cancer Patients: EORTC QLQ-BR45. Ann. Oncol..

[90] Brady M.J., Cella D.F., Mo F., Bonomi A.E., Tulsky D.S., Lloyd S.R., Deasy S., Cobleigh M., Shiomoto G. (1997). Reliability and Validity of the Functional Assessment of Cancer Therapy-Breast Quality-of-Life Instrument. J. Clin. Oncol..

[91] Ware J.E., Sherbourne C.D. (1992). The MOS 36-Item Short-Form Health Survey (SF-36). I. Conceptual Framework and Item Selection. Med. Care.

[92] Jianu D.M., Marin A. (2022). Invited Discussion on: Evaluation of Chlorhexidine Concentration on the Skin After Preoperative Surgical Site Preparation in Breast Surgery-A Randomized Controlled Trial. Aesthetic Plast. Surg..

[93] Destrebecq V., Lieveke A., Berghmans T., Paesmans M., Sculier J.-P., Meert A.-P. (2016). Are Intensive Cares Worthwhile for Breast Cancer Patients: The Experience of an Oncological ICU. Front. Med..

[94] Moscetti L., Sperduti I., Frassoldati A., Musolino A., Nasso C., Toss A., Omarini C., Dominici M., Piacentini F. (2021). Quality of Life of Therapies for Hormone Receptor Positive Advanced/Metastatic Breast Cancer: Regulatory Aspects and Clinical Impact in Europe. Breast.

[95] Hamer J., McDonald R., Zhang L., Verma S., Leahey A., Ecclestone C., Bedard G., Pulenzas N., Bhatia A., Chow R. (2017). Quality of Life (QOL) and Symptom Burden (SB) in Patients with Breast Cancer. Support. Care Cancer.

[96] Biskup E., Cai F., Vetter M., Marsch S. (2017). Oncological Patients in the Intensive Care Unit: Prognosis, Decision-Making, Therapies and End-of-Life Care. Swiss Med. Wkly..

[97] O’Shaughnessy J. (2005). Extending Survival with Chemotherapy in Metastatic Breast Cancer. Oncologist.

[98] Reshma I.A., Franchet C., Gaspard M., Ionescu R.T., Mothe J., Cussat-Blanc S., Luga H., Brousset P. (2022). Finding a Suitable Class Distribution for Building Histological Images Datasets Used in Deep Model Training—The Case of Cancer Detection. J. Digit. Imaging.

[99] Murray M.J., Coursin D.B. (1993). Multiple Organ Dysfunction Syndrome. Yale J. Biol. Med..

